# Sustainable Conversion of Waste PET into Porous Activated Carbon for Efficient Cu^2+^ Elimination from Aqueous Solution

**DOI:** 10.1021/acsomega.4c10226

**Published:** 2025-04-14

**Authors:** Jia-Yin Lin, Jun-Ren Shi, Fu-Chen Liu, Chih-Ying Wang, Fan-Wei Liu, Chi-Ming Lin

**Affiliations:** † Semiconductor and Green Technology Program, Academy of Circular Economy, National Chung Hsing University, Taichung 402, Taiwan; ‡ Industrial and Smart Technology Program, Academy of Circular Economy, National Chung Hsing University, Taichung 402, Taiwan; § Department of Environmental Engineering and Science, Chia Nan University of Pharmacy and Science, Tainan 71710, Taiwan

## Abstract

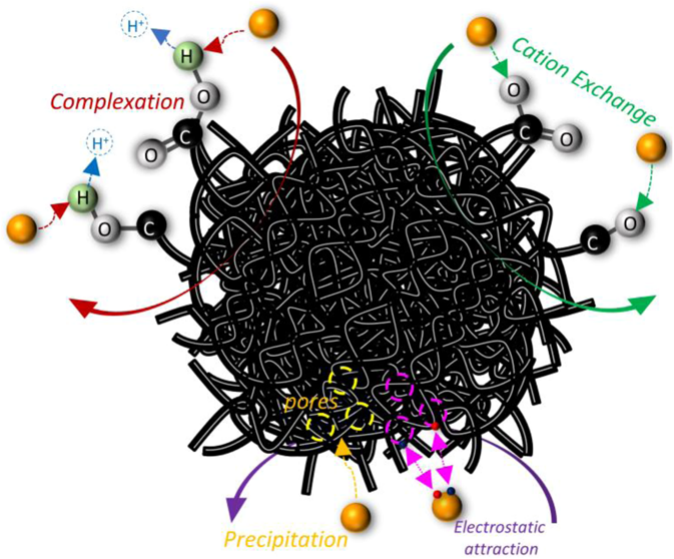

Heavy metal pollutants, such as Cu^2+^, pose significant environmental and health risks due to their toxicity and persistence in water systems. Simultaneously, the increasing accumulation of waste poly(ethylene terephthalate) (PET) bottles represents a growing environmental challenge, contributing to plastic pollution. This study addresses both issues by converting waste PET bottles into porous activated carbon (APC) via pyrolysis, creating an efficient and sustainable adsorbent for Cu^2+^ removal from aqueous solutions. The APC materials were thoroughly characterized by SEM, BET, and XPS analyses, revealing a highly porous structure and abundant oxygen-containing functional groups, which enhance Cu^2+^ adsorption. The adsorption process was determined to be spontaneous, with a low activation energy of 7.47 kJ/mol, indicating a favorable and energy-efficient adsorption mechanism. Among the APC samples, APC-800 exhibited the best performance, achieving a Cu^2+^ removal efficiency of 99.30% and a maximum adsorption capacity of 5.85 mg/g. Recyclability tests confirmed the material’s durability, maintaining over 96% efficiency during the first three cycles, with a slight decline in later cycles. This study demonstrates a dual environmental benefit: mitigating plastic waste by repurposing PET bottles and providing an effective solution for heavy metal pollution, aligning with circular economy principles, and promoting sustainability in environmental management.

## 1 Introduction

Heavy metal contamination of water resources resulting from various industrial activities is a significant environmental concern.^1^ The global decline in potable water availability is substantially intensified by the presence of heavy metal pollutants.^2^ While heavy metals like copper naturally occur in the Earth’s crust, their concentrations in the environment have escalated due to increased human usage,^3^ leading to higher levels of metal contaminants in both terrestrial ecosystems and freshwater habitats.^4^ Metals such as lead, arsenic, mercury, and copper are particularly problematic because of their extensive applications across numerous industries.^5^

Among them, copper is one of the most critical heavy metal pollutants.^6^ It is widely used in sectors including metal processing, plastic manufacturing, construction, and the production of electrical and electronic devices.^7^ Copper contamination in water bodies can stem from anthropogenic sources such as mining activities, metal production, and domestic waste discharge.^8^ The release of copper-rich wastewater into the environment poses serious risks to ecosystems, environmental health, and human well-being.^9^ Since industrial processes often generate wastewater with high copper concentrations, it is essential to treat this water to reduce copper levels before environmental release.^10^ Regulatory agencies like the United States Environmental Protection Agency (USEPA) and the World Health Organization (WHO) have set maximum allowable concentrations of copper in drinking water at 1.3 mg/L and 2.0 mg/L, respectively.^11^

In recent years, the increasing accumulation of waste polyethylene terephthalate (PET) has become a significant crisis to both humanity and the environment.^12^ Over the past decades, there has been a sharp increase in plastic production and pollution, resulting in a severe plastic waste problem that has caused more damage than previously anticipated.^13^ The immense amounts of discarded PET not only disrupt ecosystems but also pose potential risks to human health through the food chain.^14,15^ Therefore, finding effective ways to manage and repurpose waste PET to mitigate its environmental impact has become an urgent global challenge.^16,17^

However, it is worth mentioning that PET stands out among plastics due to its higher fixed carbon content of approximately 12%.^18,19^ This implies that the solid byproducts generated from the pyrolysis of PET under oxygen-limited conditions are superior carbonaceous substrates relative to those derived from other plastics.^20^ Common methods to utilize PET waste are mechanical recycling, pyrolysis, and chemical transformation.^21−23^ Among these methods, pyrolysis offers several advantages for the treatment of PET waste. Pyrolysis is a thermal decomposition process that breaks down PET polymers into smaller molecules in the absence of oxygen.^24^ Specifically, the char produced from PET pyrolysis has a high fixed carbon content and, after activation, a significantly increased specific surface area.^25−27^ This makes it an excellent carbonaceous material for applications like pollutant adsorption, including the removal of heavy metals from water.^28−30^ Additionally, pyrolysis can handle mixed or contaminated PET waste that is unsuitable for mechanical recycling, providing a more flexible and efficient waste management solution.^31^

In this work, a one-step pyrolysis process with the addition of potassium hydroxide (KOH) was employed to convert waste PET into high-yield carbon materials with a high specific surface area.^32−34^ The introduction of KOH acts as an activating agent during pyrolysis, promoting the formation of a porous carbon structure. Under a nitrogen atmosphere, KOH undergoes an etching treatment on the carbon material, generating gases such as CO_2_ and H_2_O, which facilitate the development of pores and significantly increase the surface area.^35−37^ The resulting activated carbon exhibits excellent properties for the adsorption of heavy metal ions, specifically copper.^38,39^ This method not only provides an efficient way to recycle PET waste but also offers a sustainable solution for the removal of copper from contaminated water sources. By transforming plastic waste into valuable resources, this approach aligns with the Sustainable Development Goals (SDGs), particularly SDG 6: Clean Water and Sanitation and SDG 12: Responsible Consumption and Production.^40^ It supports the principles of a circular economy by closing the loop on plastic waste, reducing environmental pollution, and promoting the sustainable management and efficient use of natural resources.^41^

## 2 Experiments

### 2.1 Reagents and Chemicals

The PET in this study was obtained from wastewater bottles. The chemicals used in this work were available technical-grade materials and were used as obtained without further purification or dilution. Potassium hydroxide (KOH), copper sulfate (CuSO_4_), copper chloride (CuCl_2_), and copper nitrate (Cu(NO_3_)_2_) were purchased from Emperor Chemical (Taiwan). Deionized (DI) water was used for the aqueous solutions.

### 2.2 Preparation and Characterization of Activated Carbon

**Figure 1:**
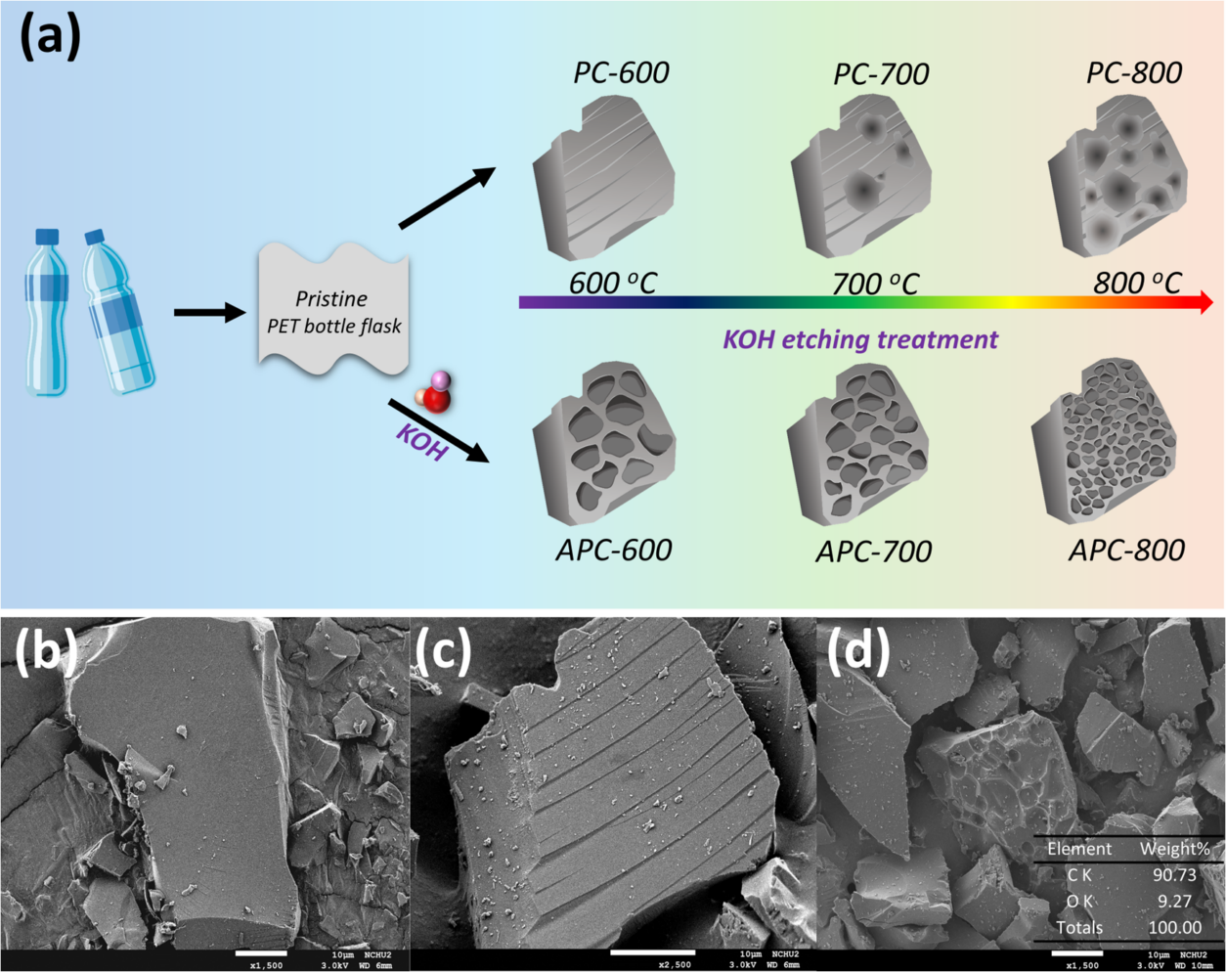
(a) Synthesis scheme for PET-derived carbon materials; SEM images for (b) PC-600, (c) PC-700, and (d) PC-800.

In this study, activated carbon was prepared through a chemical activation method, as illustrated in Figure 1. Initially, 2 g of PET was thoroughly mixed with 4 g of KOH (PET/KOH = 1:2). The mixture was placed in a ceramic container, which was then placed in a quartz tube. The quartz tube was placed in a tube furnace, and the sample was heated under a nitrogen atmosphere at a flow rate of 100 mL min^–1^ at a heating rate of 10 °C min^–1^ to three different temperatures: 600, 700, and 800 °C. Once the target temperature was achieved, the samples were maintained at that temperature for 1 h. After cooling to room temperature, the obtained products were immersed in hydrochloric acid solution for 24 h and washed with deionized water until neutral. The samples were then dried in an oven at 80 °C for 24 h and were named APC-600, APC-700, and APC-800 according to the heating temperatures. For comparison, PET was also carbonized at the same three temperatures without chemical activation, producing samples referred to as PC-600, PC-700, and PC-800. The detailed operating conditions and yields are summarized in Table S1. In this work, morphologies and internal structures of all as-prepared materials were examined by scanning electron microscopy (SEM) and transmission electron microscopy (TEM) (JEOL, Japan). Thermogravimetric analysis (TGA) of all as-prepared materials was measured by a TGA analyzer (TGA 4000, PerkinElmer). The specific surface area and pore size distribution were analyzed by using a gas adsorption analyzer (NOVAtouch, Anton Paar, Austria). Additionally, a Fourier-transform infrared (FTIR) spectrophotometer (Thermo Scientific Nicolet iS5 FT-IR) has been conducted to identify the chemical composition of materials. To verify the proposed scheme, Raman spectroscopy (MRI, PTT, Taiwan) and X-ray photoelectron spectroscopy (XPS) (PHI 5000 ULVAC-PHI, Japan) were conducted to investigate the valence states of active surface elements.

### 2.3 Batch Adsorption Test for Cu^2+^ from Aqueous Solution

Typically, CuSO_4_ was used to prepare Cu^2+^ ion solutions with an initial concentration of 10 mg L^–1^ for batch adsorption experiments. The experiments were conducted with a fixed volume of 100 mL in a 250 mL beaker, stirred at 250 rpm at room temperature. To evaluate the effects of various removal parameters, this work was conducted on contact time (0–120 min), adsorbent dosage (0.01–0.2 g), reaction temperature (30–50 °C), pH value (2–6), and different Cu^2+^ ion salts. The pH of the solutions was adjusted by using 0.2 N HCl or 0.2 N NaOH solutions. After the adsorption experiments, the concentration of Cu^2+^ ions was determined using ICP-MS, and the removal efficiencies of the different activated carbon series were evaluated. The removal efficiency of the activated carbon was calculated using eq 1.

The Cu^2+^ ion removal efficiency of the activated carbon was calculated by
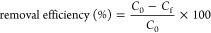
1

where *C*_0_ represents the initial concentration, and *C*_f_ represents the final concentration of the Cu^2+^ ion solutions before and after removal over activated carbons, respectively.

## 3 Results and Discussion

### 3.1 Characterization of PC and APC

The morphologies and nanostructures of the as-prepared materials in this work were examined by SEM and TEM. The waste PET is shown in Figure S1a, which exhibited a smooth surface, and the EDS data showed a significant presence of carbon (62.74%) and oxygen (35.97%), consistent with the PET structure, along with a small amount of chlorine (1.29%), likely from impurities or additives. The pyrolyzed carbon materials (PC-600, PC-700, and PC-800) are displayed in Figure 1b–d, which increasingly roughened and fractured surfaces as the pyrolysis temperature increased. The EDS analysis of PC-800 (Figure S1b) showed a significant increase in the carbon content to 90.73% and a decrease in oxygen to 9.27%, indicating effective carbonization. The reduction in oxygen and higher carbon content demonstrates that elevated pyrolysis temperatures enhance carbonization efficiency, producing materials with a higher carbon-to-oxygen ratio typical of carbonaceous structures. This change indicated structural breakdown and carbonization during heat treatment. As the temperature increased from 600 to 800 °C, the SEM images of PC-600, PC-700, and PC-800 showed more pronounced fractures and the formation of layered structures, suggesting enhanced carbonization and structural modification at higher temperatures.

**Figure 2:**
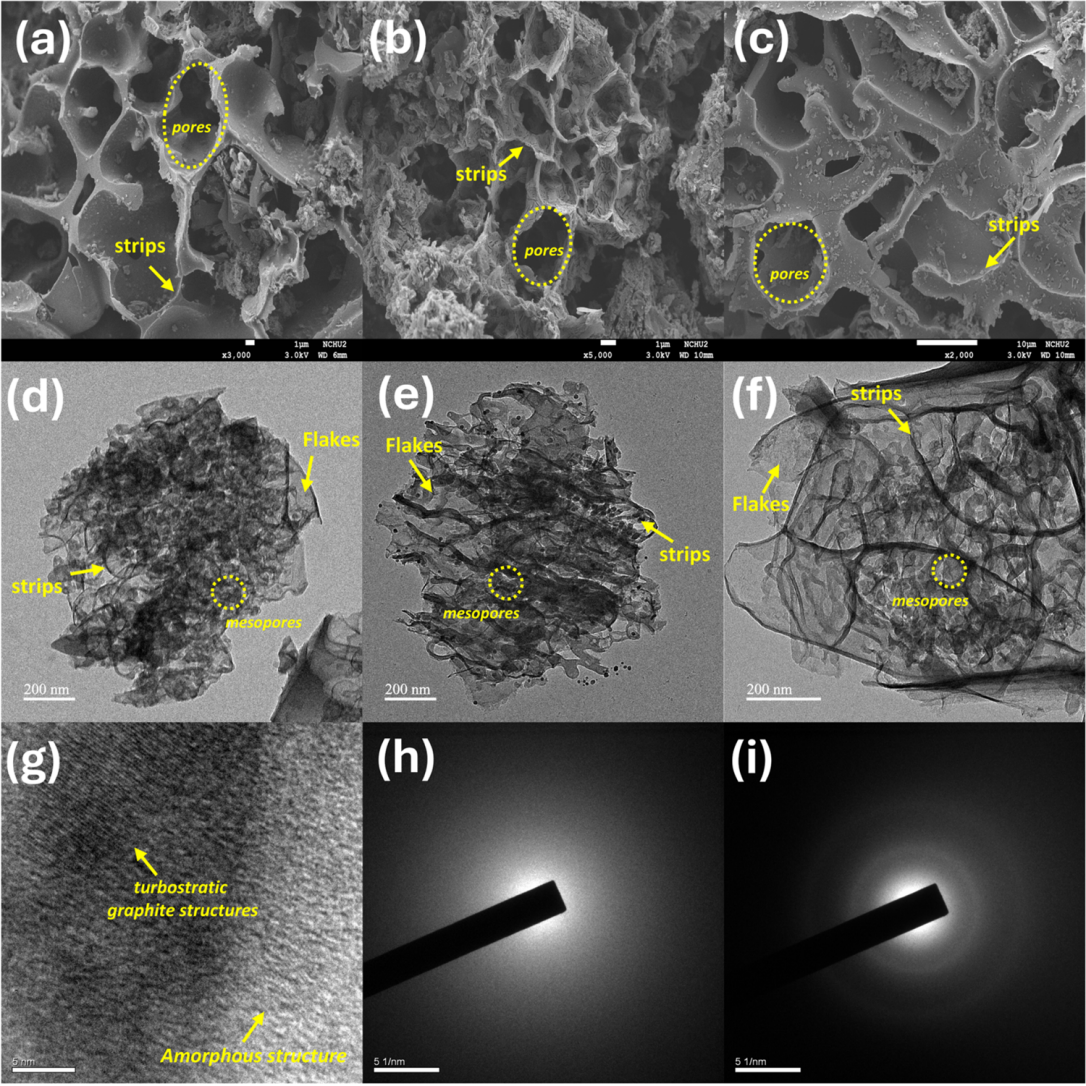
SEM images of (a) APC-600, (b) APC-700, and (c) APC-800; TEM images of (d) APC-600, (e) APC-700, and (f) APC-800; HR-TEM image of (g) APC-800 and the corresponding SAED of (h) amorphous structures and (i) graphite structures.

In contrast, Figure 2 exhibits the SEM images of APC-600, APC-700, and APC-800, revealing a significant morphological transformation compared with the PCs. The APC samples demonstrated the presence of pores and interconnected networks, indicative of the effective chemical activation by KOH. When PET was mixed with KOH and subjected to pyrolysis, KOH underwent decomposition and subsequent reactions with the carbon matrix of PET. These reactions included the oxidation of carbon by potassium compounds, leading to the removal of carbon atoms in the form of gaseous byproducts. Figure S2 supports this observation through EDS spectra, showing the elemental composition changes during activation. For APC-600, the carbon content is highest (87.90 wt %), with minimal oxygen (11.98 wt %) and potassium (0.04 wt %), reflecting limited activation. APC-700 and APC-800 exhibit increased oxygen and potassium contents, with APC-800 reaching 15.13 wt % oxygen and 14.10 wt % potassium, indicating more extensive activation and pore formation at higher temperatures. Additionally, during the etching treatment under a nitrogen atmosphere, KOH facilitated the generation of gases, such as CO_2_ and H_2_O, further contributing to pore formation and the increase in surface area.

This etching effect created a network of pores throughout the carbon structure. KOH facilitated the breakdown of the carbon framework, promoting the development of both micro- and mesopores. At elevated temperatures, KOH acted as an activating agent by intercalating into the carbon layers, causing further exfoliation and expansion of the carbon matrix. The pores and nanosized strip structures were visible in the TEM images of APC-600, APC-700, and APC-800 in Figure 2d–f, which further confirmed the porous and layered structures. The flakes observed in the TEM images were likely derived from the carbon matrix, which was exfoliated during the activation and pyrolysis processes. Furthermore, to explore the structural features, HR-TEM and SAED were analyzed and are presented in Figure 2g–h. Figure 2g shows the HR-TEM image, which highlights the predominance of amorphous structures within the biochar matrix alongside minor regions exhibiting turbostratic graphite structures. Correspondingly, the SAED pattern^42^ in Figure 2h shows diffuse rings, confirming the amorphous nature, while faint diffraction spots indicate the presence of microcrystalline graphite phases as exhibited in Figure 2i. This observation aligns with the XRD results in Figure S6, which demonstrate a broad diffraction peak characteristic of an amorphous structure, with minor sharp features suggesting a limited extent of graphitization. Using the Scherrer equation , the crystallite sizes for the carbon material were calculated based on the observed diffraction peaks.^43^ A strong peak around 2θ = 25°, corresponding to the (002) plane, yielded a crystallite size of 0.91 nm, while a weaker peak at approximately 2θ = 43°, corresponding to the (100) plane, indicated a crystallite size of 1.47 nm. These combined analyses confirm that APC-800 consists primarily of amorphous biochar with a minor proportion of graphitic structures.

**Figure 3:**
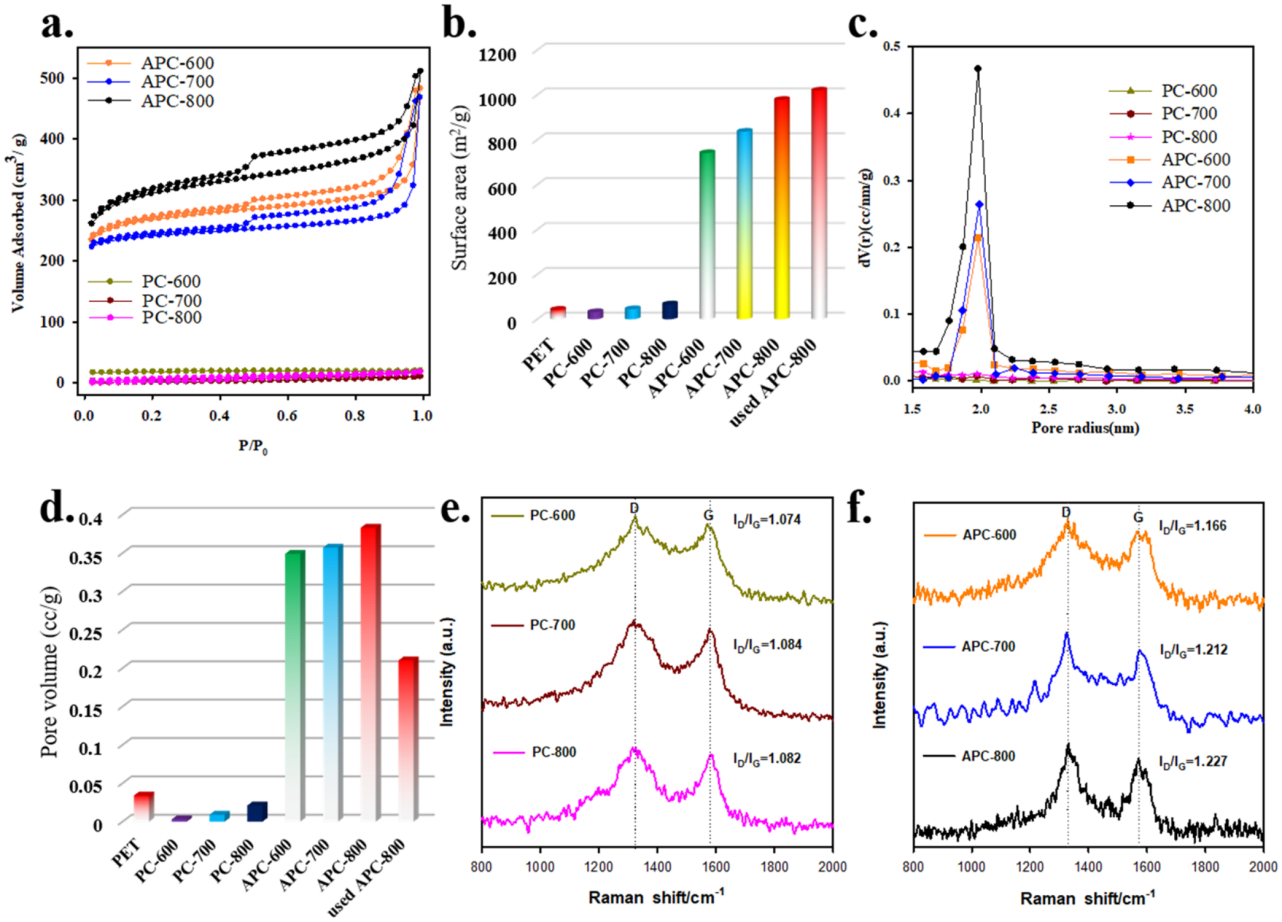
Characterization of PET and as-prepared samples: (a) N2 sorption isotherm, (b) surface area, (c) pore size distribution, and (d) pore volume; Raman spectra of (e) PC series samples and (f) APC series samples.

To characterize the textural properties of PC and APC materials, the N_2_ sorption isotherm was conducted for analysis and is presented in Figure 3a. The waste PET presents a BET surface area (*S*_BET_) of 42.95 m^2^/g. The APC samples (APC-600, APC-700, and APC-800) exhibit type IV isotherms with pronounced hysteresis loops, indicating the presence of mesopores. In contrast, the PC samples display much lower adsorption volumes, suggesting a less developed porous structure. As seen in Figure 3b and Table 1, the *S*_BET_ of APC samples is significantly higher than that of PC samples, with APC-800 reaching up to 978.55 m^2^/g compared to 66.61 m^2^/g for PC-800. This increase in surface area is attributed to the activation process involving KOH, which promotes pore formation.^44^

**Table 1:** Textural Properties of Prepared Catalysts

sample	*S*_BET_ (m^2^/g)	*V*_t_ (cc/g)	I_D_/I_G_
PET	42.95	0.034	
PC-600	32.04	0.003	1.074
PC-700	45.32	0.009	1.084
PC-800	66.61	0.021	1.082
APC-600	741.10	0.349	1.166
APC-700	836.60	0.357	1.212
APC-800	978.55	0.383	1.227

The pore size distribution in Figure 3c reveals significant differences in porosity between the PC and APC series, with APC samples (APC-600, APC-700, and APC-800) showing a notable peak in the mesoporous range (2–3 nm), especially pronounced in APC-800, indicating that higher activation temperatures facilitate greater pore formation. In contrast, PC samples (PC-600, PC-700, and PC-800) show a lower abundance of mesopores, suggesting limited porosity from pyrolysis alone. Figure 3d highlights the total pore volume differences, with APC-800 exhibiting the highest pore volume (0.383 cc/g), significantly greater than APC-600 (0.349 cc/g) and APC-700 (0.357 cc/g), while PC samples have consistently lower pore volumes (0.003–0.021 cc/g), as depicted in Table 1, underscoring the importance of chemical activation in enhancing porosity. For comparison, the waste PET, with a pore volume of 0.034 cc/g, further emphasizes the increase in porosity achieved in the APC series.

Furthermore, the yield of PC and APC after pyrolysis and activation is detailed in Table S1. The PC samples yield between 15.13 and 16.29%, whereas the APC samples have notably higher yields, particularly APC-700 with a yield of 73.65%. The increased yield of APC samples suggests that the activation process effectively retains the carbon structure while enhancing porosity.

To examine the degree of graphitization and defect levels in carbon materials, Raman spectroscopy was conducted and the resulting curves are shown in Figure 3e,f. The Raman spectra (Figure 3e,f) were used to analyze the graphitic structures of the samples. The spectra exhibit two characteristic peaks: the D-band (∼1350 cm^–1^) related to disordered carbon and the G-band (∼1590 cm^–1^) corresponding to graphitic sp^2^ carbon. The intensity ratio of these peaks (I_D_/I_G_), as presented in Table 1, provides insight into the degree of disorder and graphitization of carbon materials. For the PC series, the I_D_/I_G_ ratios range from 1.074 to 1.084, indicating a moderate level of disorder. In contrast, the APC series shows a progressively increasing I_D_/I_G_ ratio with temperature, from 1.166 for APC-600 to 1.227 for APC-800. This trend suggests that the chemical activation process, particularly at higher temperatures, introduces more defects and disorder into the carbon structure. The increase in disorder is expected due to the formation of pores and the exfoliation of the carbon matrix during activation, which is consistent with the enhanced porosity observed in the APC samples.

**Figure 4:**
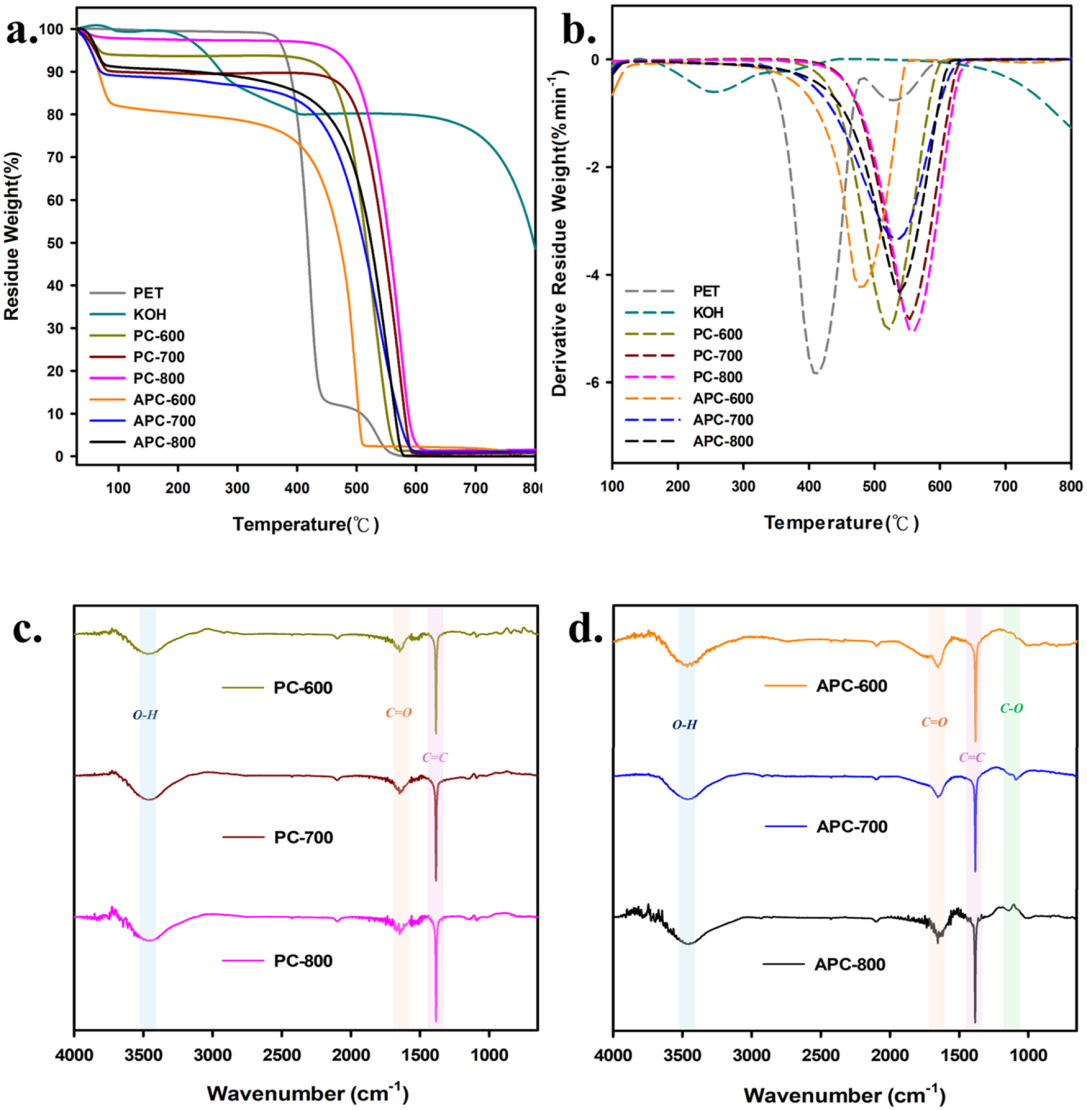
(a) TGA curves and (b) derivative curve of the PET waste and the activated carbons; FTIR spectra of (c) PC-600, PC-700, and PC-800 and (d) APC-600, APC-700, and APC-800.

Thermogravimetric analysis (TGA) was performed to assess the thermal stability and decomposition patterns of the materials, with the corresponding curves displayed in Figure 4a,b. Figure 4a presents thermogravimetric analysis (TGA) curves, illustrating the weight loss behavior of PET, KOH, PC, and APC samples as a function of temperature. PET exhibits a significant weight loss between 350 and 450 °C, indicating the decomposition of the polymer structure. KOH shows a steady weight loss between 400 and 500 °C, likely due to its decomposition or interaction with the carbon materials during activation. The PC series (PC-600, PC-700, and PC-800) displays a two-step weight loss pattern, with a minor loss at 200–300 °C (due to the removal of surface-bound groups) and a major loss between 400 and 600 °C, corresponding to the carbon framework decomposition. In contrast, the APC series (APC-600, APC-700, and APC-800) demonstrates higher thermal stability with more gradual weight loss, particularly APC-800, which can withstand higher temperatures due to the enhanced stability and porosity achieved through chemical activation.

In order to better understand the decomposition behavior and the rate of weight loss of the materials, derivative thermogravimetry (DTG) curves were analyzed. Figure 4b shows the derivative thermogravimetry (DTG) curves, highlighting the rate of weight loss as a function of the temperature. PET exhibits a sharp peak around 400 °C, corresponding to its rapid decomposition, while KOH shows a smaller peak at 400–500 °C. The PC samples have broader DTG peaks between 400 and 600 °C, indicating a slower rate of decomposition due to their increased thermal stability after pyrolysis. The APC samples, especially APC-800, exhibit DTG peaks shifted to higher temperatures, signifying greater thermal resistance. The broader and higher-temperature peaks in APC samples confirm the structural robustness and enhanced thermal stability resulting from the activation process.

To identify and analyze the surface functional groups present on the carbon materials, FTIR spectroscopy was conducted. The FTIR spectra of PC and APC samples in Figure 4c,d provide insight into the surface functional groups of the pyrolyzed and activated carbon materials. The FTIR spectra of PC-600, PC-700, and PC-800 (Figure 4c) show peaks corresponding to various functional groups. Notably, the peak around 3400 cm^–1^ indicates the presence of O–H stretching, while peaks at approximately 1600 and 1700 cm^–1^ correspond to C = C and C = O stretching vibrations, respectively. These peaks suggest the presence of hydroxyl and carbonyl groups on the surface of pyrolyzed carbon.

However, through KOH chemical activation, the FTIR spectra of APC-600, APC-700, and APC-800 (Figure 4d) show significant changes, particularly in the intensities of the oxygen-containing functional groups. The O–H stretching band around 3400 cm^–1^ becomes more pronounced in the activated carbons, indicating an increase in the number of hydroxyl groups. Similarly, the C = O stretching band around 1700 cm^–1^ shows greater intensity, suggesting a higher concentration of carbonyl groups on the surface. Additionally, the C–O stretching at approximately 1100 cm^–1^ becomes more prominent, indicating the formation of ether or ester linkages. These changes confirm that the activation process introduces more oxygen-containing functional groups, which enhance the surface chemistry of APC materials and improve their adsorption capacity for metal ions.

### 3.2 Adsorption Kinetics, Isotherms, and Thermodynamics of Cu^2+^ Removal Using Activated Carbon Materials

**Figure 5:**
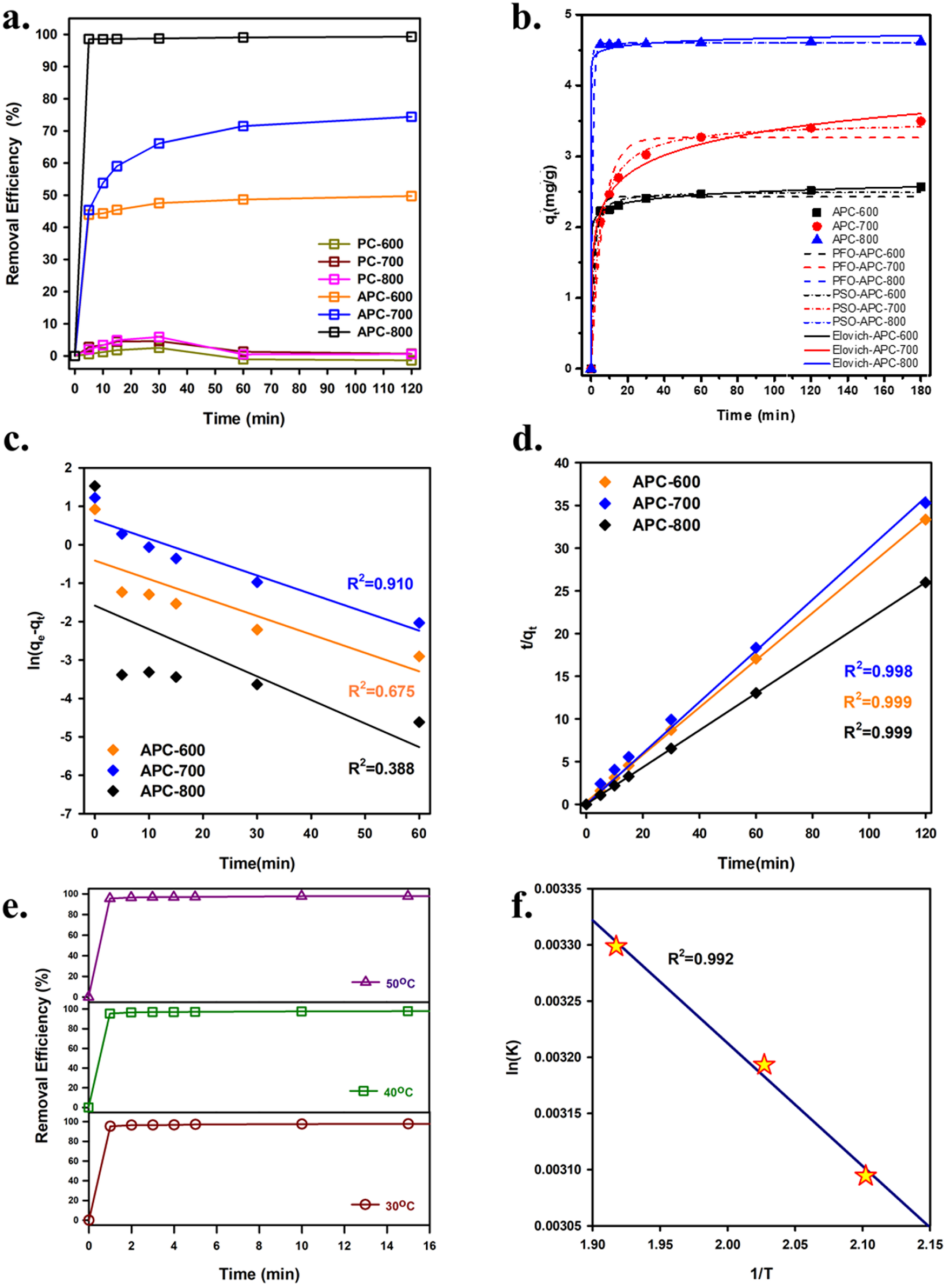
(a) Effect of contact time on Cu^2+^ removal from aqueous solution using different materials; (b) PFO, PSO, and Elovich kinetic model of APC-600, APC-700, and APC-800; (c) PFO plots; (d) PSO plots; (e) effect of temperature using APC-800; and (f) Arrhenius plots (ln *K* vs 1/T) for Cu^2+^ removal in the range of 30–50 °C.

To understand the performance of APC-600, APC-700, and APC-800 in removing Cu^2+^ ions, adsorption kinetics were evaluated, as shown in Figure 5a–d and Table 2. Figure 5a demonstrates that APC-800 exhibits the highest removal efficiency, reaching nearly 100% within 60 min, followed by APC-700 at around 70% and APC-600 at 40% after 120 min. In contrast, the PC series shows significantly lower removal efficiencies, with PC-600, PC-700, and PC-800 achieving less than 10% removal, emphasizing the importance of chemical activation in improving the adsorption performance of APC materials.

**Figure 6:**
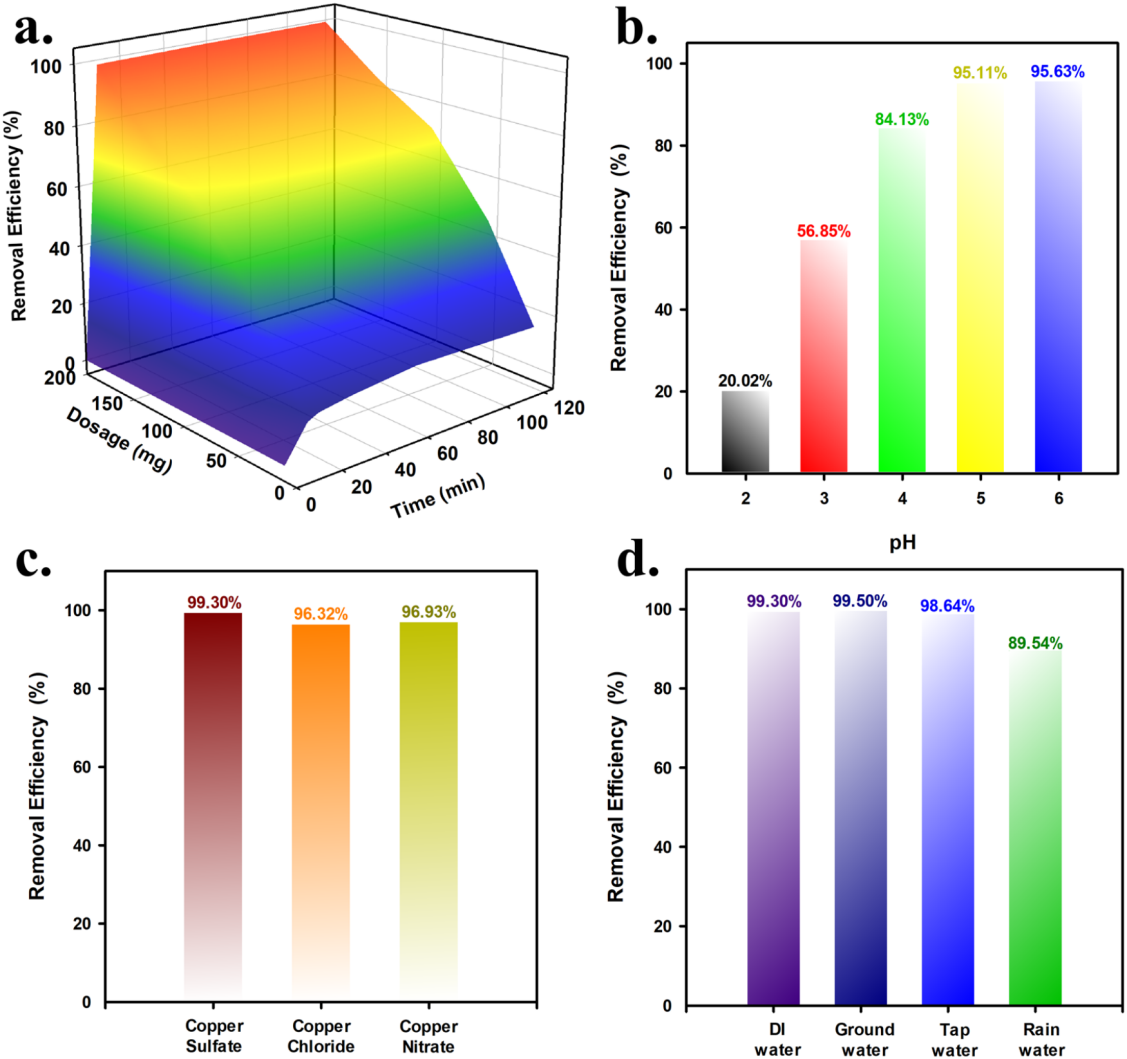
Cu^2+^ removal using APC-800 on the influence of (a) dosage, (b) pH, (c) various copper salts, and (d) water body.

Moreover, the adsorption kinetics were modeled using both pseudo-first-order and pseudo-second-order equations. Figure 5b shows that the pseudo-second-order model provides a better fit, with higher *R*^2^ values for all APC samples, especially APC-800, indicating that chemisorption is the dominant mechanism. Figures 5c and 6d further confirm this, as the linearized form of the pseudo-second-order model (Figure 5d) shows excellent correlation (*R*^2^ ≈ 0.999) for APC-600, APC-700, and APC-800, compared to lower correlation for the pseudo-first-order model (Figure 5c). The kinetic parameters in Table 1 also support this, as the pseudo-second-order adsorption capacities (q_e_(cal)) closely match the experimental values, particularly for APC-800, where the experimental adsorption capacity (q_e_(exp)) reaches 4.61 mg/g. Additionally, the rate constant (*K*_2_) for APC-800 is the highest, indicating faster adsorption kinetics, making it the most effective material for Cu^2+^ removal due to its enhanced surface properties from the activation process. Furthermore, Figure S7 presents the adsorption isotherm models, including Langmuir (Figure S7a), Freundlich (Figure S7b), and Temkin (Figure S7c), with their corresponding fitting results plotted in Figure S7d. The Langmuir model (*R*^2^ = 0.996) suggests that adsorption occurs on a homogeneous monolayer surface, whereas the Freundlich model (*R*^2^ = 0.972) indicates adsorption on a heterogeneous surface with multilayer adsorption characteristics. Additionally, the Temkin model (*R*^2^ = 0.98) accounts for the influence of adsorbate–adsorbent interactions, further supporting the mixed chemisorption–physiosorption mechanism.

Table S4 summarizes the isotherm model constants for APC-800. The Langmuir maximum adsorption capacity (q_m_) is 5.71 mg/g, with a Langmuir constant *K*_L_ of 1.26 L/mg, confirming a strong affinity for Cu^2+^ adsorption. The Freundlich constant (*K*_F_ = 4.15 mg/g) and the adsorption intensity parameter (*n* = 14.53) further suggest that APC-800 has a favorable adsorption capacity under varying concentrations. Lastly, the Temkin constants (*B*_T_ = 0.27 J/g and *K*_T_ = 1.02 × 10^7^ L/g) indicate a moderate adsorption heat, further supporting the coexistence of chemisorption and physisorption mechanisms.^45^

For the Elovich model (Figure S8a and Table 2), the adsorption behavior of APC materials varies with temperature.^46^ APC-600 exhibits stable adsorption with a slower initial rate (α = 3.71 mg/g min) and desorption constant (β = 2.37 g/mg), with a moderate fit (*R*^2^ = 0.75). APC-700 shows better performance with α = 1.75 mg/g of min and β = 1.50 g/mg, fitting well to the model (*R*^2^ = 0.92). APC-800 achieves the highest adsorption rate (α = 19.42 mg/g min), indicating rapid adsorption, though the fit is lower (*R*^2^ = 0.58). The intraparticle diffusion model (Table S5 and Figure S8b) further clarifies the mechanisms. APC-800 shows the highest initial diffusion rate (*K*_p1_ = 1.56 mg/g min^–0.5^, *R*^2^ = 0.92), while APC-600 has a slower diffusion (*K*_p1_ = 0.76 mg/g min^–0.5^). In the second stage, APC-600 has a reduced diffusion rate (*K*_p2_ = 0.03 mg/g min^–0.5^), reaching equilibrium quickly, while APC-700 (*K*_p2_ = 0.09 mg/g min^–0.5^) indicates moderate internal diffusion. APC-800s lowest *K*_p2_ (0.01 mg/g min^–0.5^) suggests greater diffusion limitations despite its strong initial diffusion.

**Table 2:** Kinetic Model Rate Constant for Cu^2+^ Using APC-600, APC-700, and APC-800 at *T* = 30 °C, *C*_0_ = 10 ppm, w = 200 mg, and *V* = 0.1 L

		pseudo-first order			pseudo-second order			Elovich		
sample	q_e_ (exp)	q_e_ (cal)	*K*_1_	*R*^2^	q_e_ (cal)	*K*_2_	*R*^2^	β (g/mg)	α (mg/g min)	*R*^2^
APC-600	3.59	3.80	0.05	0.67	3.61	0.27	0.99	2.37	3.71	0.75
APC-700	3.40	1.79	0.04	0.91	3.46	0.09	0.99	1.50	1.75	0.92
APC-800	4.61	22.4	0.06	0.38	4.61	2.62	0.99	1.47	19.42	0.58

Figure 5e shows the effect of temperature on Cu^2+^ removal efficiency using APC-800 at 30, 40, and 50 °C. The removal efficiency increases with temperature, with the highest efficiency observed at 50 °C, reaching nearly 100% removal within 5 min. At 40 and 30 °C, the efficiency remains lower, indicating that elevated temperatures accelerate the adsorption process, likely due to enhanced diffusion of Cu^2+^ ions into the porous structure of APC-800.

Furthermore, Figure 5f presents the Arrhenius plot, which is used to calculate the activation energy (*E*_a_) for Cu^2+^ removal. The linearity of the plot, with a high correlation coefficient (*R*^2^ = 0.992), indicates that the removal process follows the Arrhenius behavior. The calculated activation energy (*E*_a_ = 7.47 kJ/mol) is relatively low, suggesting that the adsorption process is energetically favorable and can occur easily with minimal energy input. Compared with previous studies (Table S2), APC-800 offers excellent adsorption efficiency with lower energy barriers. Additionally, APC-800 demonstrates a maximum adsorption capacity (*q*_max_) of 5.85 mg/g, which is comparable to or better than that of other adsorbents, further confirming its superior performance for Cu^2+^ removal.

Moreover, Table 3 presents the thermodynamic parameters for Cu^2+^ adsorption on APC-800, including the Gibbs free-energy change (Δ*G*), enthalpy change (Δ*H*), and entropy change (Δ*S*). The detailed formulas used to calculate these thermodynamic parameters are provided in the Supporting Information. The negative values of Δ*G* at all temperatures (303, 313, and 323 K) indicate the spontaneous adsorption process.^47^ As the temperature increases, the more negative Δ*G* values (from −4.832 at 303 K to −5.648 at 323 K) indicate that the spontaneity of the adsorption process becomes stronger, aligning with the enhanced removal efficiency observed at higher temperatures. Since the Δ*G* values are within the range of −20 kJ/mol, this suggests that physisorption is the dominant mechanism, involving weaker electrostatic interactions between the adsorbate and the adsorbent surface. Typically, Δ*G* values below −20 kJ/mol are indicative of physisorption, while more negative values, typically below −40 kJ/mol, would suggest chemisorption, involving stronger interactions such as charge transfer or covalent bonding.^47,48^ In this case, the moderate Δ*G* values indicate that the adsorption of Cu^2+^ onto APC-800 is primarily driven by physisorption, although the increasing spontaneity at higher temperatures suggests that the process remains efficient across the temperature range.

**Table 3:** Data of Thermodynamic Parameters for the Adsorption of Cu^2+^ on APC-800

sample	temp (K)	*E*_a_ (kJ/mol)	Δ*G* (kJ/mol)	Δ*H* (kJ/mol)	Δ*S* (J/mol K)
APC-800	303	7.47	–4.832	1.174	0.029
	313		–5.277		
	323		–5.648		

Additionally, the positive value of Δ*H* (1.174 kJ/mol) confirms that the adsorption process is endothermic,^49,50^ meaning that it absorbs heat, further supporting the observation that higher temperatures improve the adsorption performance. Finally, the positive Δ*S* values indicate increased randomness at the solid–liquid interface during the adsorption process,^51^ likely due to the release of water molecules as Cu^2+^ ions are adsorbed onto the surface of APC-800.

### 3.3 Influence of Dosage and pH on the Cu^2+^ Removal from Aqueous Solution

Since APC-800 showed the highest Cu^2+^ removal efficiency, its efficiency was further tested under different operating conditions to optimize performance. Figure 6a presents the effect of adsorbent dosage on the Cu^2+^ removal efficiency using APC-800. As the dosage increases from 10 to 200 mg, the removal efficiency improves significantly. At the lowest dosage of 10 mg, the removal efficiency is only 11.15% after 10 min, reaching 16% after 120 min. However, with a dosage of 200 mg, the removal efficiency reaches approximately 99% within 60 min, indicating a substantial increase in performance. The table shows that increasing the dosage leads to more active sites being available for Cu^2+^ adsorption, resulting in faster and more effective removal of copper ions from the solution.

Additionally, Figure 6b demonstrates the effect of pH on the Cu^2+^ removal efficiency. The removal efficiency increases as the pH increases from 2 to 6, reaching a maximum of 95% at pH 5 and 6. At lower pH levels, such as pH 2, the removal efficiency is much lower (20.02%), likely due to the competition between H^+^ ions and Cu^2+^ ions for the adsorption sites on APC-800. As the pH increases, the concentration of H^+^ ions decreases, allowing more Cu^2+^ ions to interact with the surface functional groups of APC-800, thus improving the removal efficiency.

### 3.4 Effect of Various Copper Salts, Heavy Metals, and Water Body on the Cu^2+^ Removal from Aqueous Solution

Furthermore, Figure 6c illustrates the influence of various copper salts on the Cu^2+^ removal efficiency. The removal efficiency remains consistently high across all three copper salts, copper sulfate, copper chloride, and copper nitrate, with APC-800 achieving 99.30% removal for copper sulfate, 96.32% for copper chloride, and 96.93% for copper nitrate. These results indicate that the type of copper salt has minimal effect on the adsorption efficiency of APC-800, demonstrating its versatility in treating various forms of copper contamination in aqueous solutions.

Figure 6d shows the effect of different water sources on the removal efficiency of Cu^2+^ using an APC-800. The removal efficiency remains high across different water types, including DI (deionized) water, groundwater, tap water, and rainwater. APC-800 achieves nearly identical removal efficiencies of 99.30% in DI water, 99.50% in groundwater, and 98.64% in tap water, indicating that the material performs consistently well regardless of the water source. However, the removal efficiency decreases slightly to 89.54% in rainwater, which may be due to the presence of additional dissolved substances or impurities in rainwater that can interfere with Cu^2+^ adsorption. Despite this minor decrease, the overall removal efficiency remains high, demonstrating the versatility and robustness of APC-800 in treating Cu^2+^ contamination in various water bodies. On the other hand, Figure S9shows the removal efficiency of APC-800 for Cu^2+^, Fe^3+^, and Co^2+^, achieving 99.30, 95.90, and 96.33%, respectively. These results demonstrate the strong adsorption performance and versatility of APC-800 in removing various metal ions with slightly higher efficiency for Cu^2+^, likely due to stronger interactions with the functional groups on APC-800. This highlights its potential for treating wastewater containing diverse metal contaminants.

Moreover, Figure S10a,b illustrates the effects of humic acid and NaCl on Cu^2+^ removal efficiency using APC-800. In Figure S10a, as the concentration of humic acid increases from 20 to 60 mg/L, the removal efficiency of Cu^2+^ improves from 80.54 to 95.16%. This suggests that humic acid may enhance Cu^2+^ adsorption by forming complexes with Cu^2+^, facilitating its interaction with the functional groups on APC-800. Figure S10b demonstrates the impact of ionic strength on Cu^2+^ adsorption by introducing 10, 25, and 50 ppm of NaCl. The results indicate that although Cl^–^ can interact with Cu^2+^ to form CuCl_2_ complexes, potentially reducing free Cu^2+^ ions available for adsorption, the removal efficiency remains above 85%, confirming that APC-800 maintains a high adsorption capacity even in saline environments. This suggests that the dominant adsorption mechanism is not solely electrostatic attraction but also involves chemical interactions, making APC-800 applicable to various water sources.

### 3.5 The Proposed Reaction Mechanism for Cu^2+^ Removal Using APC-800

**Figure 7:**
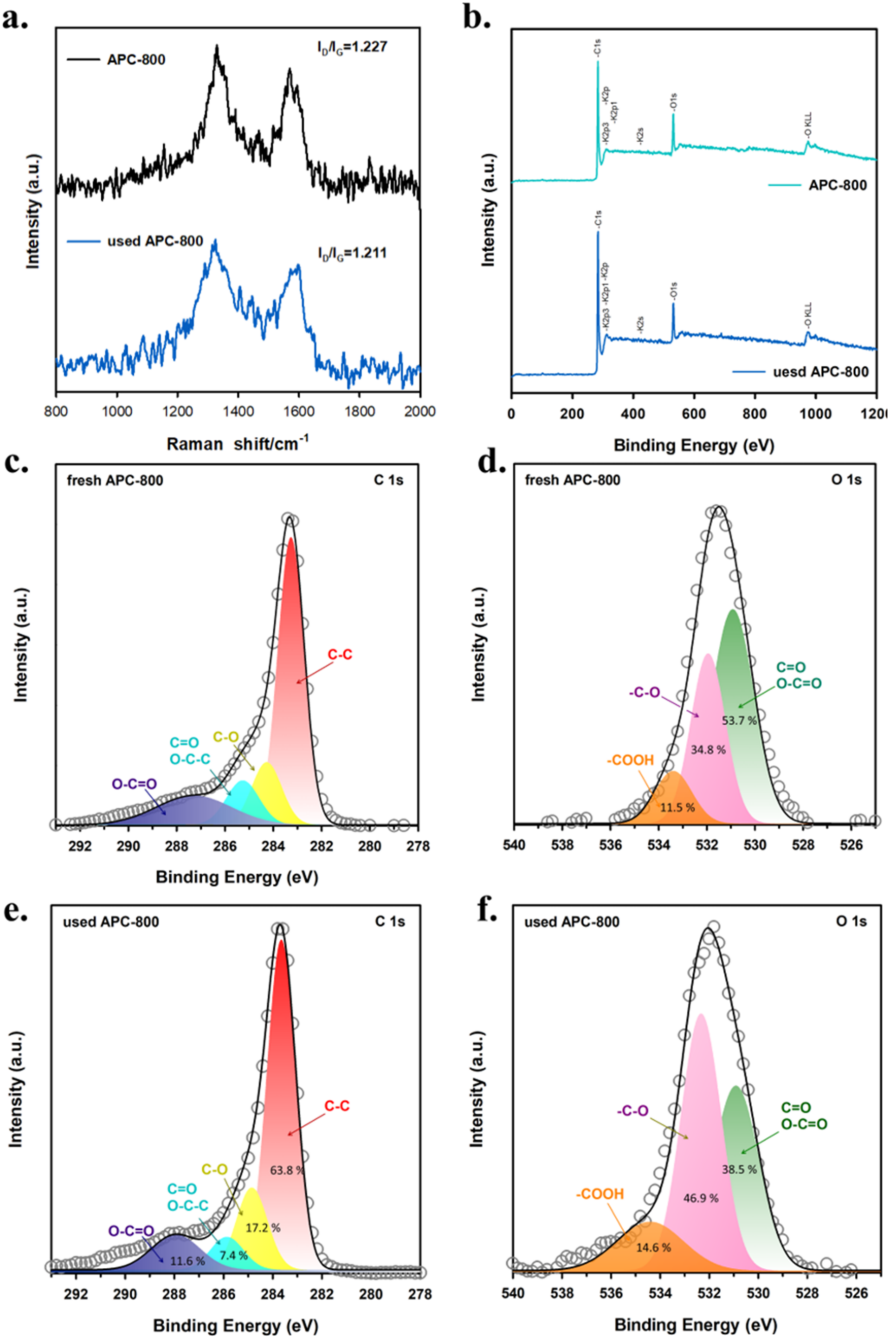
(a) Raman spectra and (b) XPS survey spectra of fresh APC-800 and used APC-800; XPS analysis of fresh APC-800: (c) C 1S and (d) O 1s; XPS analysis of used APC-800: (e) C 1S and (f) O 1s.

To investigate the surface chemical state and structure of APC-800, XPS and Raman analyses were conducted, as presented in Figure 7. Figure 7a shows the Raman spectra of fresh and used APC-800. The two characteristic peaks around 1350 and 1590 cm^–1^ correspond to the D-band (related to defects in the carbon structure) and the G-band (indicative of graphitic sp^2^ carbon), respectively. The intensity ratio of these bands (I_D_/I_G_) provides insight into the degree of disorder and graphitization in the carbon material. The I_D_/I_G_ ratio for fresh APC-800 is 1.227, slightly higher than that of used APC-800 (1.211), suggesting that the adsorption process slightly reduces the number of defects, possibly due to interactions between Cu^2+^ ions and the carbon surface.

Figure 7b–f presents the XPS spectra, which further clarify the chemical composition of APC-800 before and after Cu^2+^ adsorption. The XPS spectra of fresh APC-800 in the C 1s region (Figure 7c) reveal distinct peaks corresponding to different carbon bonds: C–C (58.9%), C, O (14.2%), C, C = O (11.0%), and C = O (16.7%). These oxygen-containing functional groups, especially C–O, O–C = O, and C = O, provide active sites for Cu^2+^ adsorption. Similarly, the O 1s spectrum (Figure 7d) confirms the presence of oxygen-containing groups, showing peaks for C = O/O/C = O (53.7%), −COOH (11.5%), and C–O (34.8%). The carboxyl (−COOH) and hydroxyl groups are particularly important for chelating and complexing with Cu^2+^ ions, enhancing adsorption onto the carbon surface.

After Cu^2+^ adsorption, changes in the XPS spectra of APC-800 are observed, providing insights into the interaction between Cu^2+^ and the adsorbent’s surface. The C 1s spectrum of used APC-800 (Figure 7e) shows an increase in the relative intensity of C–C bonds to 63.8%, while the percentages of C–O (7.4%), O–C = O (11.6%), and C = O (17.2%) exhibit variations, indicating possible interactions between Cu^2+^ ions and oxygen-containing functional groups.^52,53^ In the O 1s spectrum (Figure 7f), a noticeable increase in the – COOH group content (from 11.5 to 14.6%) is observed, along with shifts in the distribution of C = O/O–C = O (38.5%) and C–O (46.9%) species.^54^ These changes suggest that oxygen-containing functional groups on the APC-800 surface, particularly carboxyl and hydroxyl groups, actively participate in the binding of Cu^2+^ ions through mechanisms such as complexation or ion exchange.^55^ On the other hand, the N_2_ sorption isotherm and pore size distribution of used APC-800 (Figure S5a,b) confirm the preservation of its mesoporous structure, with a type IV isotherm and a prominent pore size peak around 2–3 nm. However, the reduced nitrogen adsorption volume and a slight decrease in the BJH pore volume suggest partial pore blockage by adsorbed Cu^2+^ ions. The BET analysis (Table S3) shows a minor increase in surface area, likely due to pore restructuring during adsorption, while the Raman analysis reveals a slight reduction in the I_D_/I_G_ ratio, indicating a decreased defect density. These results demonstrate that APC-800 retains its structural robustness and mesoporous characteristics, with minor modifications after Cu^2+^ adsorption.

Additionally, Figure S4 shows the XPS analysis of the Cu 2p spectrum for used APC-800, further confirming the adsorption of Cu^2+^ ions. The Cu 2p spectrum exhibits two prominent peaks at binding energies of 932.7 and 952.5 eV, corresponding to Cu 2p_3_/_2_ and Cu 2p_1_/_2_, respectively. These peaks are characteristic of Cu^2+^, verifying that copper ions were successfully adsorbed onto the APC-800 surface. The presence of a satellite peak (sat.) around 944 eV is indicative of the typical shakeup feature associated with Cu^2+^ species, confirming the oxidation state and the successful adsorption of copper onto the activated carbon. This further supports the conclusion that APC-800 effectively adsorbs Cu^2+^ ions with oxygen-containing functional groups playing a key role in the adsorption process.

**Figure 8:**
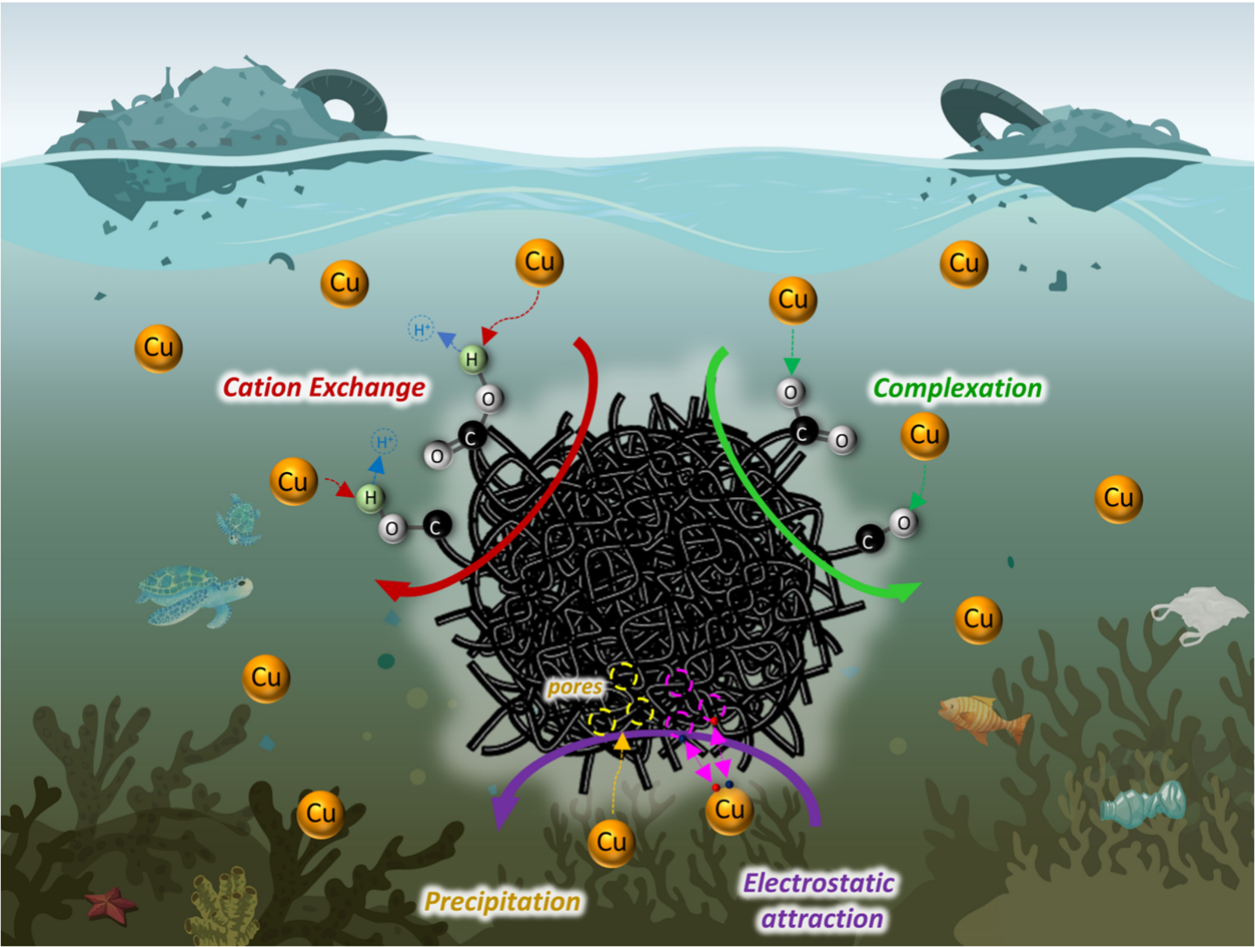
Proposed adsorption mechanism for the removal of Cu^2+^ using APC-800.

Based on characterization and experimental data, including Raman and XPS analyses, the proposed mechanism for Cu^2+^ adsorption onto APC-800 involves both physical and chemical interactions facilitated by the material’s high surface area, porosity, and oxygen-containing functional groups, as depicted in the proposed adsorption mechanism (Figure 8).

Initially, when Cu^2+^ ions come into contact with the surface of APC-800 in an aqueous solution, they diffuse toward the carbon material. The KOH activation process enhances the surface area and pore structure of APC-800, providing abundant adsorption sites for Cu^2+^. This large surface area allows for increased physical adsorption, where Cu^2+^ ions are drawn toward the carbon matrix. Once Cu^2+^ ions are near the surface, chemical adsorption occurs through the surface complexation. The oxygen-containing functional groups, such as hydroxyl (−OH), carbonyl (C = O), and carboxyl (−COOH) groups, act as ligands that bind Cu^2+^ ions. This complexation happens as the lone electron pairs on the oxygen atoms form coordinate bonds with the Cu^2+^ ions, stabilizing them on the carbon surface. This is confirmed by the XPS data, which show an increase in the content of oxygen species after Cu^2+^ adsorption, indicating the active participation of these functional groups in binding the metal ions. Additionally, ion exchange plays a significant role in the adsorption process. Protons (H^+^) or other cations previously attached to the functional groups can be replaced by Cu^2+^ ions, allowing for an effective exchange mechanism.^56^ This exchange process is supported by the shifts in the XPS 1s spectra, where the distribution of oxygen species, particularly carboxyl and hydroxyl groups, changes after Cu^2+^ adsorption.

After the Cu^2+^ ions are adsorbed onto the APC-800 surface, they are further stabilized by the interaction with the carbon matrix. The increased presence of oxygen-containing groups (such as C = O and −COOH) after adsorption suggests that these groups act as anchoring sites, ensuring that the Cu^2+^ ions remain strongly bound to the surface, preventing desorption.^57^ The small change in the Raman I_D_/I_G_ ratio between fresh and used APC-800 indicates that the structural integrity of the material remains largely intact after Cu^2+^ adsorption, suggesting that APC-800 may be regenerated and reused for further adsorption cycles.

### 3.6 Recyclability of APC-800

**Figure 9:**
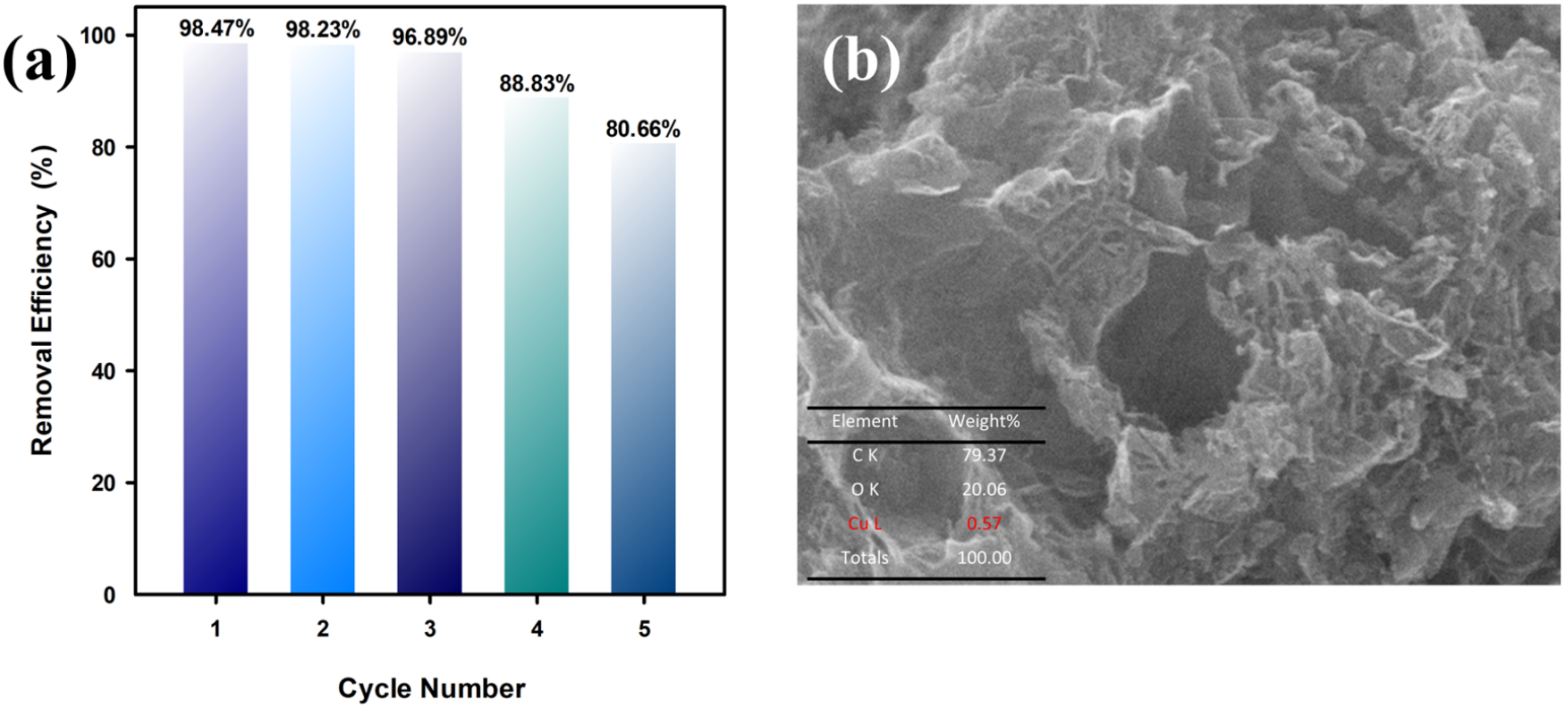
(a) Recyclability of APC-800 and (b) SEM image of used APC-800.

Figure 9a illustrates the recyclability of APC-800 for Cu^2+^ adsorption over five cycles. The removal efficiency remains high during the first three cycles, at 98.47, 98.23, and 96.89%, respectively. However, a decline in efficiency occurs in the fourth and fifth cycles, dropping to 88.83 and 80.66%, respectively. This suggests that while APC-800 is reusable, its adsorption capacity decreases slightly with each cycle, likely due to the saturation of active sites or partial degradation of surface functional groups. Furthermore, Figure S10c,d further supports these findings by demonstrating the recyclability of APC-800 for Cu^2+^ removal in tap water (Figure S10c) and groundwater (Figure S10d) over five cycles. In tap water, APC-800 maintains high removal efficiencies of 99.99, 99.99, and 98.80% in the first three cycles, with a slight decline to 87.73 and 96.70% in the fourth and fifth cycles, respectively. Similarly, in groundwater, the removal efficiency remains high, starting at 92.85% in the first cycle and maintaining at 97.51% in the fifth cycle. These results highlight the robustness of APC-800 in real-world applications, demonstrating its potential for effective and repeated use in diverse water sources.

Figures 9b and S3 show SEM images and EDS spectra of APC-800 after 5 adsorption cycles, revealing a rougher, more irregular surface as a result of Cu^2+^ adsorption. The EDS analysis confirms the presence of 0.57% Cu on the surface, indicating the successful adsorption of copper ions. Additionally, high concentrations of carbon (79.37%) and oxygen (20.06%) suggest that the oxygen-containing functional groups, which play a critical role in Cu^2+^ binding, remain active even after multiple cycles. Notably, the absence of potassium (K) in the EDS spectrum may be attributed to the washing process during regeneration, which likely removed residual K from the initial activation step. Despite a slight reduction in performance over the five cycles, APC-800 retains a robust structure and significant adsorption capacity, as confirmed by both the SEM and EDS results.

## 4 Conclusions

This study demonstrates the effectiveness of APC-800, an activated carbon material derived from PET waste, for the removal of Cu^2+^ ions from aqueous solutions. APC-800 achieved a high removal efficiency of up to 99.30% and a maximum adsorption capacity (q_e_) of 5.85 mg/g. The high surface area (978.55 m^2^/g), porosity, and oxygen-containing functional groups, confirmed by Raman and XPS analyses, significantly enhance its adsorption performance. The adsorption follows pseudo-second-order kinetics, with a low activation energy (*E*_a_) of 7.47 kJ/mol, indicating an energetically favorable process. APC-800 also shows strong recyclability, maintaining over 96% efficiency for the first three cycles, though efficiency drops to 80.66% by the fifth cycle. SEM and EDS confirm Cu^2+^ adsorption and the stability of the carbon matrix. Overall, APC-800 provides a sustainable solution for heavy metal removal, aligning with circular economy principles, and offers promise for environmental pollution control applications due to its high efficiency, reusability, and ease of preparation.

## Data Availability

All data supporting the findings of this study are included in the published article and its Supporting Information. And data can be provided by the corresponding author upon reasonable request.

## Supporting Information Available

Experimental details, XPS spectra of functional groups before and after Cu^2+^ adsorption, BET analysis, SEM images of APC-800, supporting figures illustrating adsorption kinetics and isotherms, and additional tables summarizing adsorption performance across various water matrices (PDF)
