# Supplementary material for: Sustainable Conversion of Waste PET into Porous Activated Carbon for Efficient Cu2+ Elimination from Aqueous Solution
Source: ACS Omega. 2025 Apr 14;10(15):14994–5008. doi: 10.1021/acsomega.4c10226 (PMC12019729; doi:10.1021/acsomega.4c10226)
Supplement: Supplementary file 1 — ao4c10226_si_001.pdf [file ao4c10226_si_001.pdf]

Supplementary Material

For

Sustainable Conversion of Waste PET into Porous Activated Carbon for Efficient Cu^{2+} Elimination from aqueous solution

Jia-Yin Lin[ab*], *Jun-Ren Shi*[b], *Fu-Chen Liu*[c], *Chih-Ying Wang*[a], *Fan-Wei Liu*[a], *and Chi-Ming Lin*[b]

[a] Semiconductor and Green Technology Program, Academy of Circular Economy, National Chung Hsing University, Taichung, TW 402

[b] Industrial and Smart Technology Program, Academy of Circular Economy, National Chung Hsing University, Taichung, TW 402

[c] Department of Environmental Engineering and Science, Chia Nan University of Pharmacy and Science, Tainan, TW 71710

*Corresponding Authors. E-mail addresses: joylin7@dragon.nchu.edu.tw (*Jia-Yin Lin*)

Thermodynamic parameters for Cu^{2+} adsorption:

1. Gibbs Free Energy Change (ΔG): The Gibbs free energy change is calculated using the following equation:

$$\Delta G = -RT \ln K_c$$

Where:

- ΔG = Gibbs free energy change (kJ/mol)
- R = Universal gas constant (8.314 J/mol·K)
- T = Temperature in Kelvin (K)
- K_c = Equilibrium constant (dimensionless), calculated from the adsorption data.

2. Enthalpy Change (ΔH) and Entropy Change (ΔS): The van 't Hoff equation is used to determine the enthalpy and entropy changes:

$$\ln K_c = \left(\frac{-\Delta H}{R}\right) \cdot \frac{1}{T} + \frac{\Delta S}{R}$$

This equation is often rearranged into a linear form for plotting:

$$\ln K_c = \left(\frac{-\Delta H}{R}\right) \cdot \frac{1}{T} + \frac{\Delta S}{R}$$

From the slope and intercept of a plot of $\ln K_c$ versus $\frac{1}{T}$:

- Slope = $-\frac{\Delta H}{R}$, from which ΔH can be calculated.

- Intercept = $\frac{\Delta S}{R}$, from which ΔS can be calculated.

3. Relationship between Gibbs Free Energy, Enthalpy, and Entropy: Once ΔH and ΔS are known, Gibbs free energy can also be confirmed using this relation:

$$\Delta G = -\Delta H - T\Delta S$$

Where:

- ΔG = Gibbs free energy change (kJ/mol)

- ΔH = Enthalpy change (kJ/mol)
- T = Temperature in Kelvin (K)
- ΔS = Entropy change (kJ/mol·K)

Table S1. Detailed operation condition and yield of prepared materials

PC series				
Temperature(^oC)	Gas flows (mL/min)	Isothermal time (hr)	Sample name	Yield (%)
600	100	1	PC-600	16.29
700	100	1	PC-700	15.40
800	100	1	PC-800	15.13
APC series				
Temperature (^oC)	Gas flows (mL/min)	Isothermal time (hr)	Sample name	Yield (%)
600	100	1	APC-600	69.06
700	100	1	APC-700	73.65
800	100	1	APC-800	58.18

Table S2. Comparison of adsorption capacity for copper and activation energy value using various materials

Metal ion	Adsorbent	q_{max} (mg/g)	E_a	References
Cu^{2+}	APC-800	5.85	7.47	This study
	t-MWCNT	12.34	27.19	[1]
	bagasse fly ash	2.26	2.73	[2]
	COS-rGO	18.70	137.80	[3]
	manganese oxide coated zeolite (MOCZ)	1.56	9.72	[4]
	modified activated carbon	1.92	59.96	[5]
	Spherical AC	3.68	7.41	[6]
	Arenga pinnata Merr fruit shell (APMFS) waste	6.54	22.64	[7]

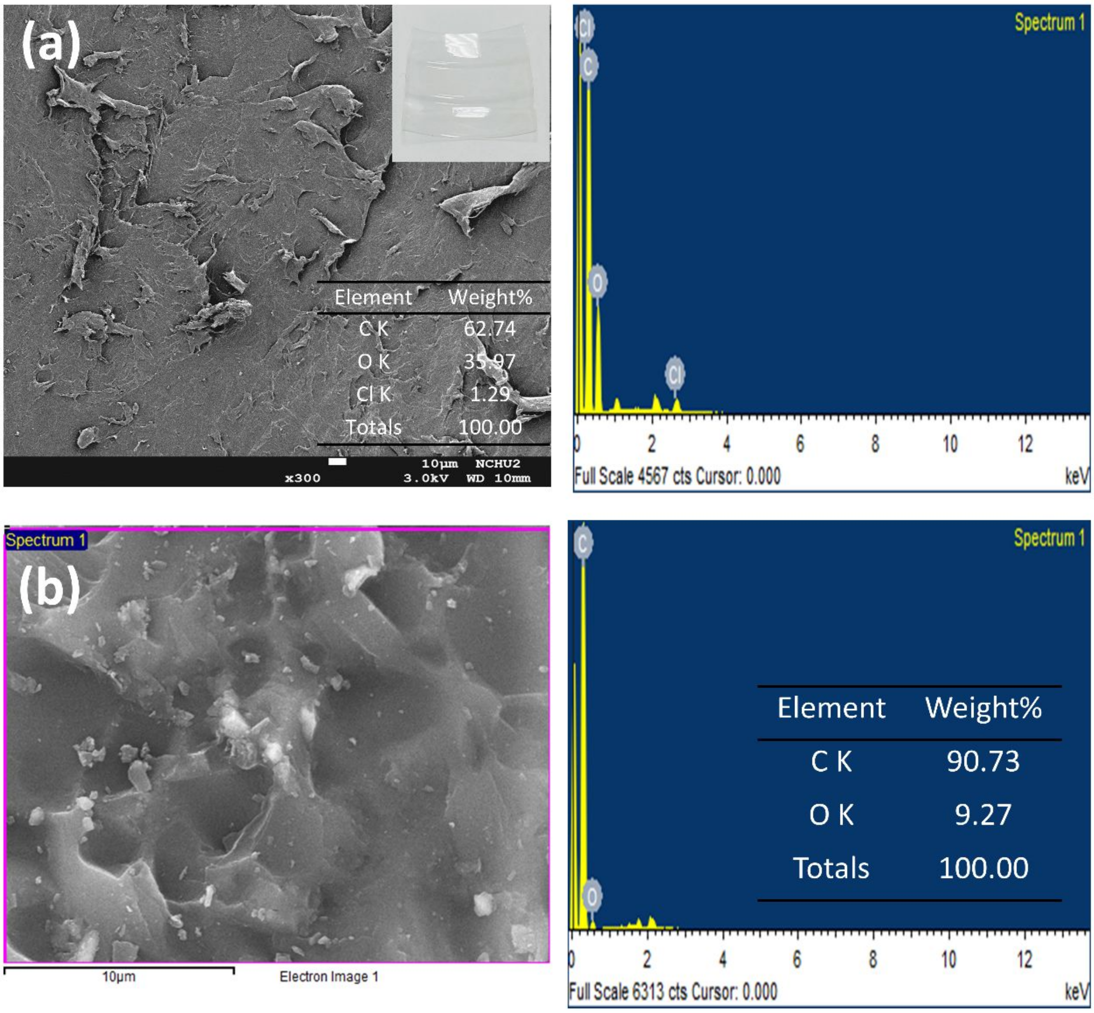

Fig. S1. SEM images and EDS spectra for the (a) waste PET, (b) PC-800

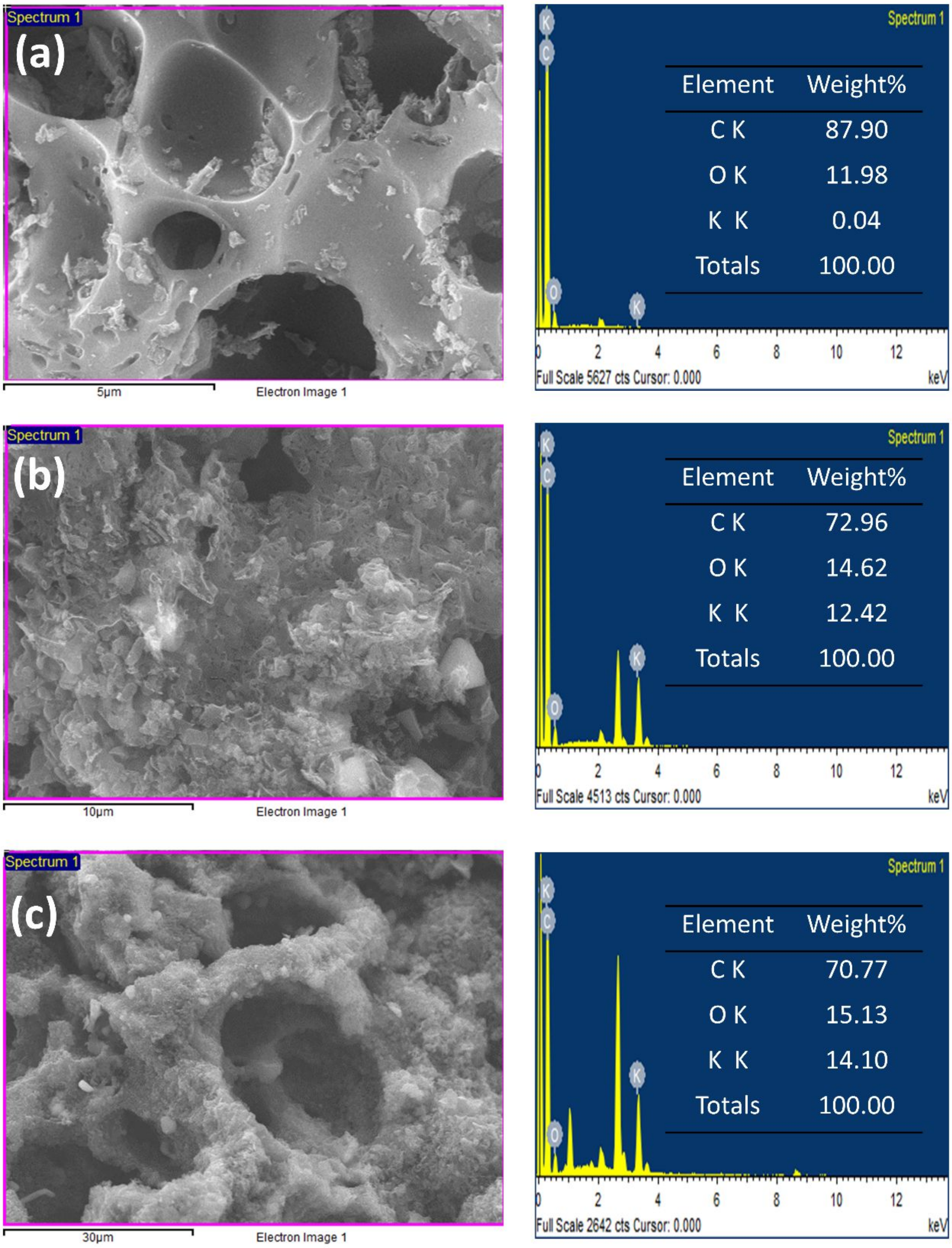

Fig. S2. SEM images and EDS spectra for the (a) APC-600, (b) APC-700 and (c) APC-800

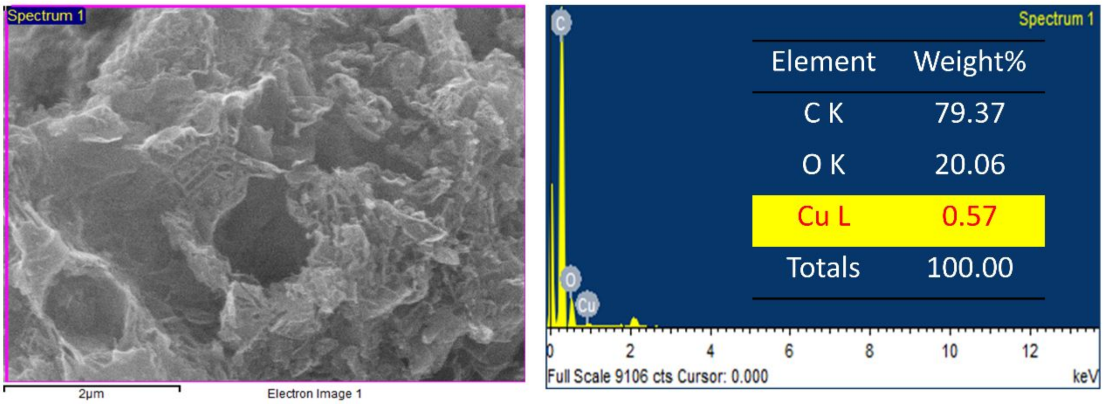

Fig. S3. SEM images and EDS spectra for the used APC-800

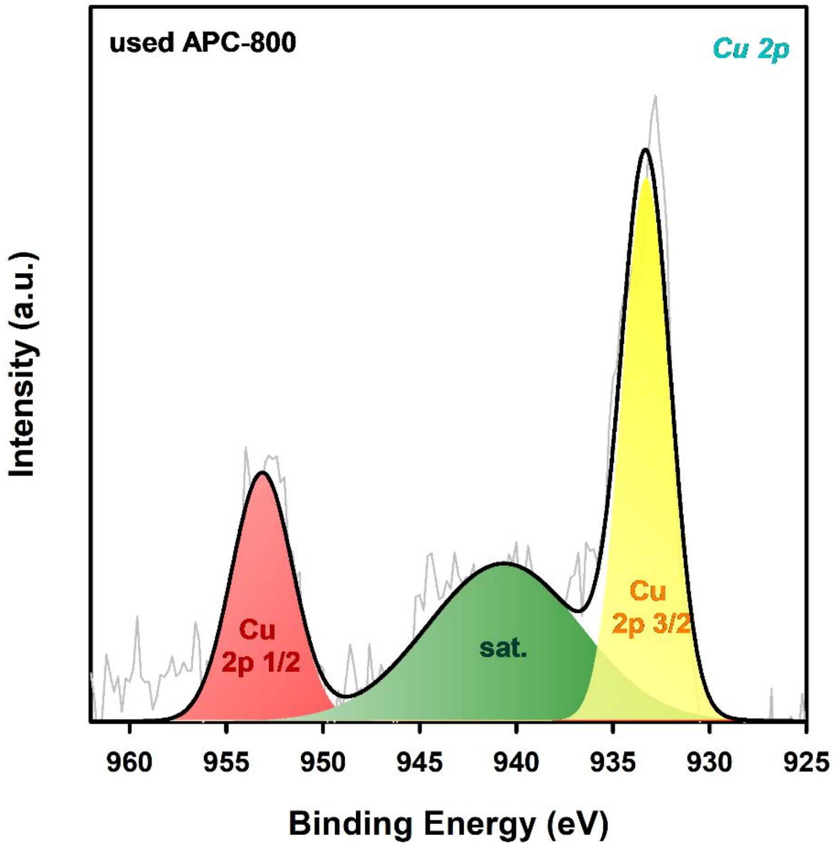

Fig. S4. XPS analysis of used APC-800 Cu 2p

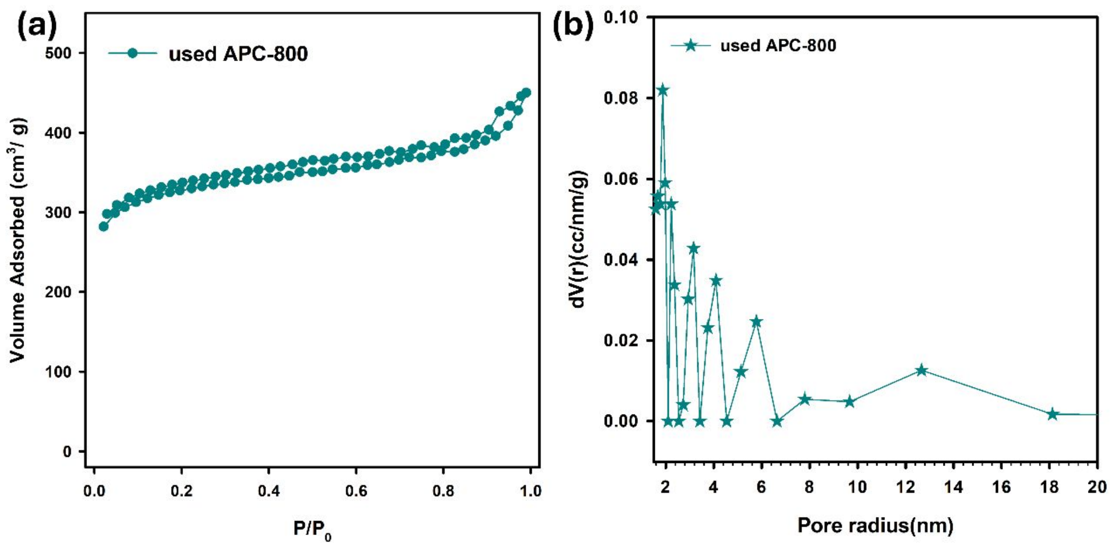

Fig. S5. (a) N_2 sorption isotherm; (b) pore size distribution of used APC-800

Table S3. Textural properties of used APC-800

Sample	S_{BET} (m^2/g)	V_t (cc/g)	I_D/I_G
Used APC-800	1020.09	0.210	1.211

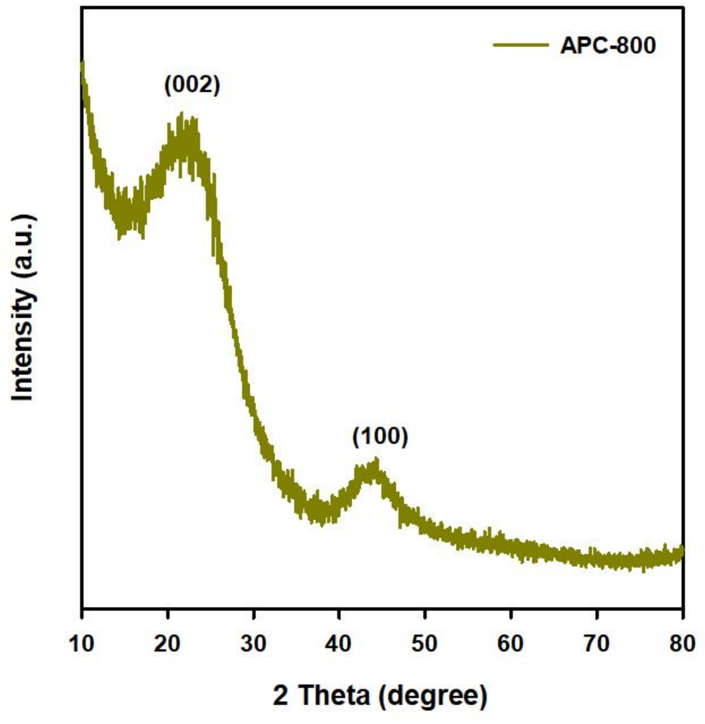

Fig. S6. XRD analysis of APC-800

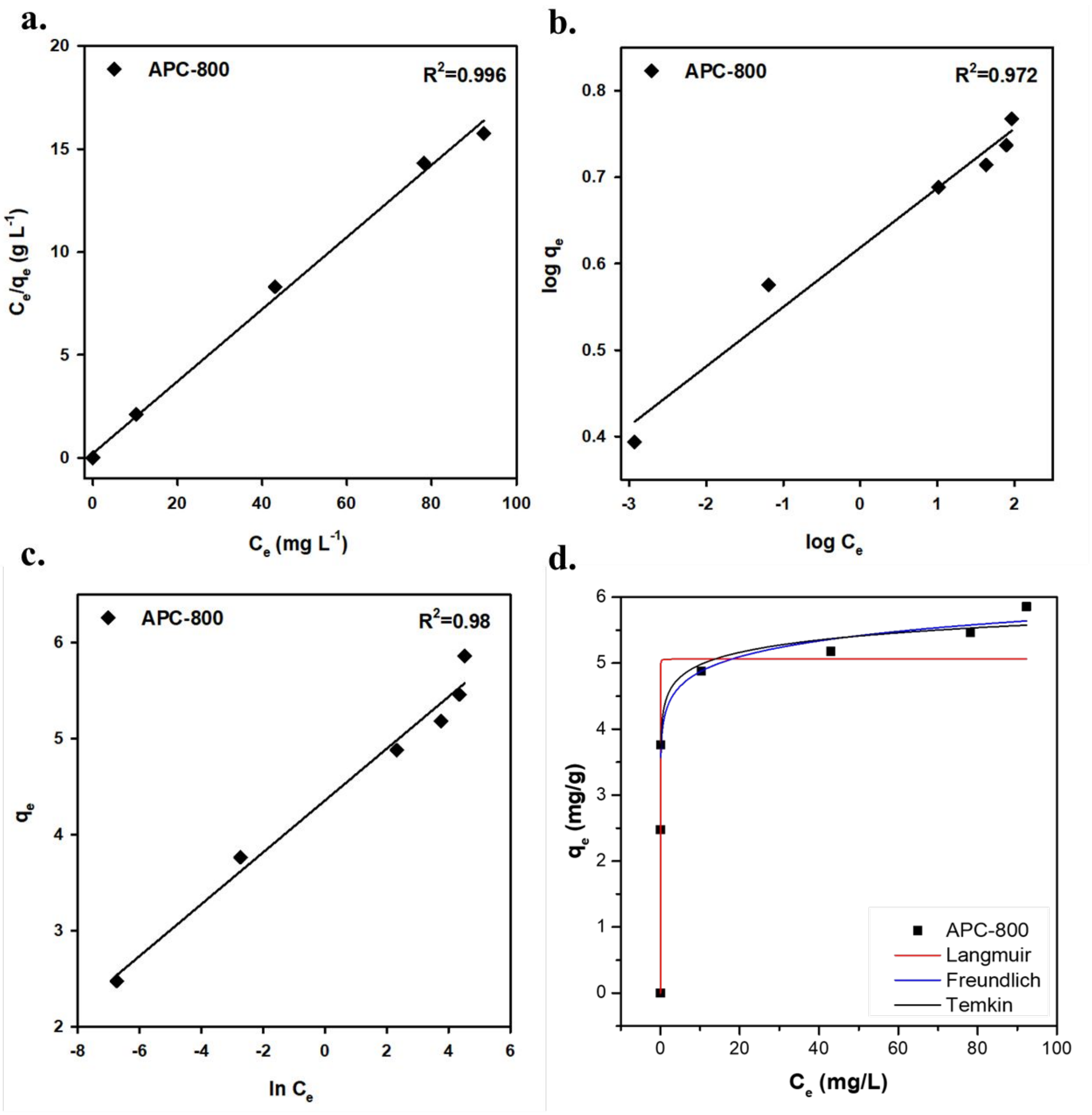

Fig. S7. (a) Langmuir isotherm plots, (b) Freundlich isotherm plots, (c) Temkin isotherm plots, (d) Langmuir, Freundlich and Temkin isotherm model of APC-800

Table S4. Langmuir, Freundlich and Temkin isotherm model constant for Cu^{2+} using APC-800 at T=30 ^{o}C, w= 200mg, V= 0.1 L

**Sample**	**Langmuir**			**Freundlich**			**Temkin**		
	q_m (mg/g)	K_L (L/mg)	R^2	n	K_F (mg/g)	R^2	B_T (J/g)	K_T (L/g)	R^2
APC-800	5.71	1.26	0.99	14.53	4.15	0.97	0.27	1.02×10^7	0.98

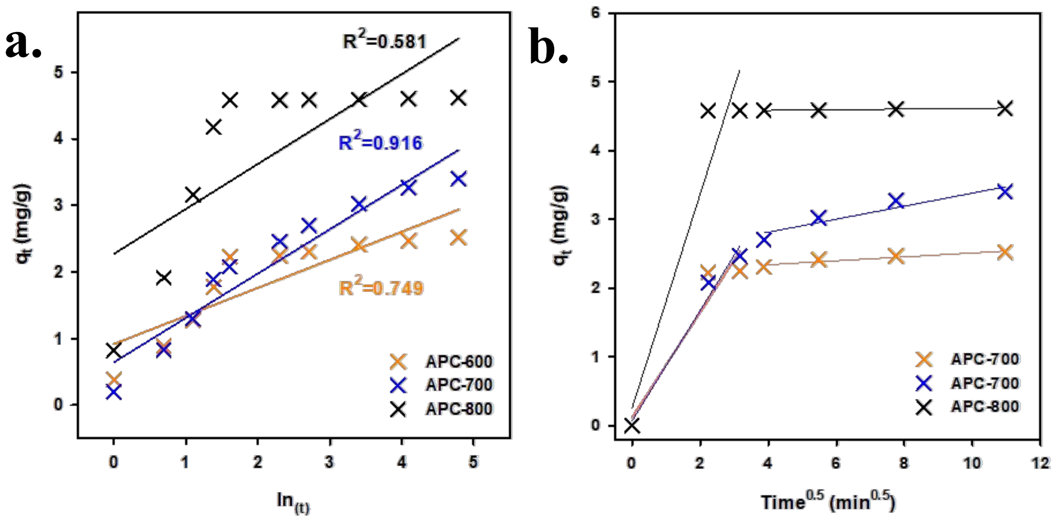

Fig. S8. (a) Elovich plots, (b) intraparticle diffusion plots

Table S5. The intraparticle diffusion model constant for Cu^{2+} using APC-600, APC-700 and APC-800 at T=30 °C, C_0=10 ppm, w= 200mg, V= 0.1 L

Sample	First section			Second section			Without separation		
	C	K_{p1}	R^2	C	K_{p2}	R^2	C	K_p	R^2
APC-600	0.12	0.76	0.92	2.22	0.03	0.90	1.24	0.16	0.44
APC-700	0.06	0.81	0.98	2.43	0.09	0.89	1.16	0.26	0.69
APC-800	0.25	1.56	0.92	4.56	0.01	0.97	2.61	0.27	0.34

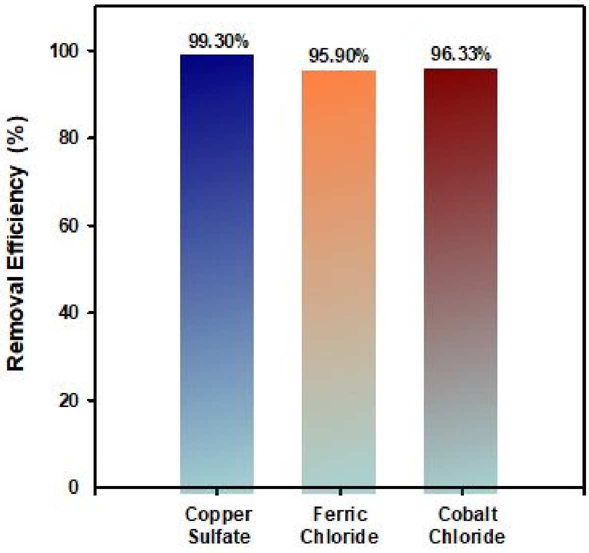

Fig. S9. Cu^{2+}, Fe^{3+}, and Co^{2+} removal efficiency using APC-800.

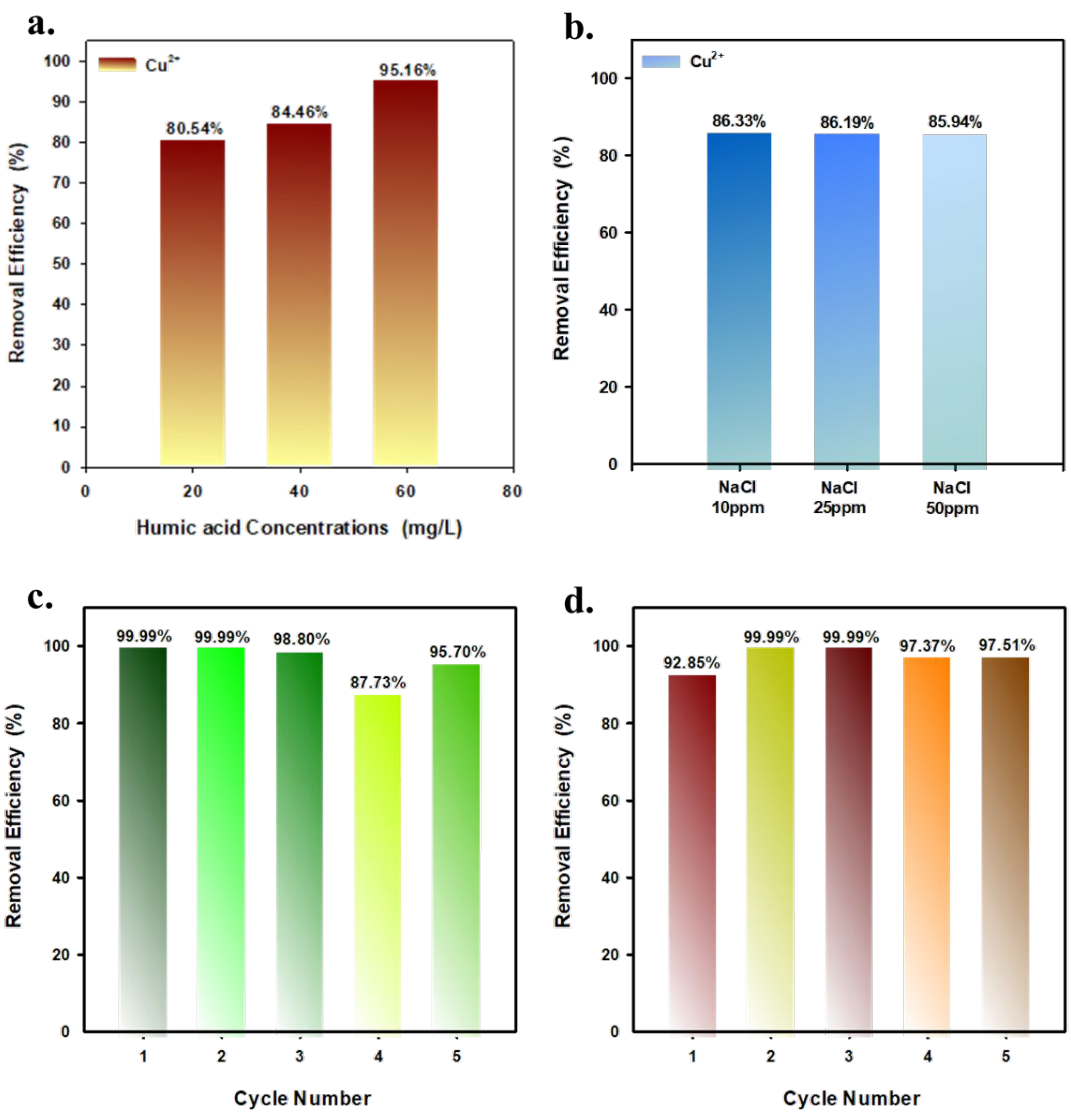

Fig. S10. Cu^{2+} removal using APC-800 on influence of (a) humic acid, (b) NaCl, recyclability of APC-800 under (c) tap water, (d) groundwater

Reference

(1) Mobasherpour, I.; Salahi, E.; Ebrahimi, M. Thermodynamics and kinetics of adsorption of Cu(II) from aqueous solutions onto multi-walled carbon nanotubes. *Journal of Saudi Chemical Society* **2014**, *18* (6), 792-801. DOI: 10.1016/j.jscs.2011.09.006.

(2) Wibowo, H.; Ketwong, T.; Cholwatthanatanakorn, N.; Ding, L.; Areeprasert, C. Production of bagasse fly ash-derived CO(2) adsorbent by physical activation and by nitrogen-functionalization using hydrothermal treatment. *Waste Manag* **2024**, *177*, 66-75. DOI: 10.1016/j.wasman.2024.01.029.

(3) Jiang, S.; Do, H.; Yusuf, A.; Xiao, Z.; Wang, C.; Li, J.; Sun, Y.; Ren, Y.; He, J. Camellia oleifera shell–reduced graphene oxide for adsorption of copper(II). *Materials Chemistry and Physics* **2024**, *314*. DOI: 10.1016/j.matchemphys.2023.128818.

(4) Zou, W.; Han, R.; Chen, Z.; Jinghua, Z.; Shi, J. Kinetic study of adsorption of Cu(II) and Pb(II) from aqueous solutions using manganese oxide coated zeolite in batch mode. *Colloids and Surfaces A: Physicochemical and Engineering Aspects* **2006**, *279* (1-3), 238-246. DOI: 10.1016/j.colsurfa.2006.01.008.

(5) Shirendev, N.; Bat-Amgalan, M.; Aleksandr, A.; Gunchin, B.; Yunden, G. *Cu(II), Pb(II) and Cr(VI) Adsorption on the Modified Activated Carbon*; 2021. DOI: 10.2991/ahcps.k.211004.021.

(6) Yin, C.; Zhang, Y.; Tao, Y.; Zhu, X. Competitive adsorption behavior and adsorption mechanism of limestone and activated carbon in polymetallic acid mine water treatment. *Sci Rep* **2024**, *14* (1), 23561. DOI: 10.1038/s41598-024-74240-8.

(7) Said, S. D.; Muslim, A.; Yahya, A.; Razali, N.; Angesta, Q. Y.; Irmayani, I.; Hadibarata, T.; Kadri, A. Adsorption of Cu(II) from Aqueous Solution on Sonicated Activated Carbon Prepared from Arenga Pinnata Merr Fruit Shell Waste: Isotherm, Kinetic and Thermodynamic Studies. *Environmental Research, Engineering and Management* **2023**, *79* (4), 112-123. DOI: 10.5755/j01.erem.79.4.32708.
